# Evidence for Digital Health Tools Designed to Support the Triage of Musculoskeletal Conditions in Primary, Urgent, and Emergency Care Settings: Scoping Review

**DOI:** 10.2196/81578

**Published:** 2026-01-14

**Authors:** Linda K Truong, James G Wrightson, Raphaël Vincent, Eunice Lui, Jamon L Couch, Ellen Wang, Cobie Starcevich, Dean Giustini, Alex Haagaard, Elena Lopatina, Niels van Berkel, Michael Skovdal Rathleff, Clare L Ardern

**Affiliations:** 1 Centre for Aging SMART, University of British Columbia, Vancouver, BC, Canada; 2 Department of Physical Therapy, Faculty of Medicine, University of British Columbia, 13737 96 Avenue, Surrey, BC, V3V 0C6, Canada, 1 604 822 4519; 3 Department of Family Practice, Faculty of Medicine, University of British Columbia, Vancouver, BC, Canada; 4 School of Rehabilitation, Faculty of Medicine, Université de Montréal, Montreal, QC, Canada; 5 Hôpital Maisonneuve-Rosemont Research Center, Université de Montréal Affiliated Research Center, Montreal, QC, Canada; 6 Centre for Interdisciplinary Research in Rehabilitation of Greater Montreal, Institut Universitaire sur la Réadaptation en Déficience Physique de Montréal, Montreal, QC, Canada; 7 La Trobe Sport and Exercise Medicine Research Centre, La Trobe University, Melbourne, Australia; 8 Arthritis Research Canada, Vancouver, BC, Canada; 9 School of Allied Health, Faculty of Health Sciences, Curtin University, Perth, Australia; 10 Physiotherapy Department, Rockingham General Hospital, South Metropolitan Health Service, Perth, Australia; 11 Biomedical Branch Library, University of British Columbia, Vancouver, BC, Canada; 12 Pain BC, Vancouver, BC, Canada; 13 University of Calgary, Calgary, AB, Canada; 14 Primary Care Alberta, Calgary, AB, Canada; 15 Department of Computer Science, Aalborg University, Aalborg, Denmark; 16 Department of Health Science and Technology, Faculty of Medicine, Aalborg University, Aalborg, Denmark; 17 Center for General Practice at Aalborg University, Aalborg, Denmark

**Keywords:** musculoskeletal, digital health, emergency department, health system, triage, PRISMA, Preferred Reporting Items for Systematic Reviews and Meta-Analyses

## Abstract

**Background:**

The digital health research field is growing rapidly, and a summary of the available digital tools for triaging musculoskeletal conditions is needed. Effective and safe digital triage tools for musculoskeletal conditions could support patients and clinicians in making informed care decisions and may contribute to reducing emergency department overcrowding and health care costs.

**Objective:**

The aim of the study is to identify and describe digital health tools for use by adults to triage musculoskeletal conditions across primary, urgent, or emergency care settings.

**Methods:**

Our scoping review was conducted following the Johanna Briggs Institute recommendations for scoping reviews and Arksey and O’Malley’s framework. Systematic searches in MEDLINE (OVID), Embase (OVID), CENTRAL (OVID), CINAHL (EBSCO), Compendex, Web of Science, OpenGrey, Google Scholar, arXiv, medRxiv, and an extensive gray literature search were conducted with a librarian scientist from inception to September 18, 2025. Studies had to recruit adults (aged 18 years and older) with musculoskeletal conditions that identified a digital health tool designed to triage or diagnose in primary, urgent, or emergency care settings and report primary data to be included. In total, 2 reviewer pairs independently screened abstracts and full-text papers. Relevant data were extracted in duplicate, and results were summarized descriptively.

**Results:**

The search yielded 5695 records, and we screened 189 full-text papers. In total, 34 studies (n=37,509 patients) met the inclusion criteria. The most common musculoskeletal conditions reported were rheumatoid or inflammatory arthritis (13/34, 38%). In total, 19 (19/34, 56%) studies reported on symptom checkers, 13 (13/34, 38%) studies on triage or diagnosis tools, and 2 (2/34, 6%) were studies of diagnostic predictor tools. There were 16 unique digital health tools. A total of 2 tools were built for triaging musculoskeletal conditions and were not publicly available outside the UK National Health Service. Most tools were generic tools designed to screen for general health problems, including musculoskeletal conditions. The most common approach to evaluating performance (eg, accuracy) of the tools was to compare the concordance of the tool to a clinician diagnosis or triage recommendation. Sensitivity and specificity ranged from 39% to 91% and 23% to 80%, respectively. The reported accuracy of the included tools ranged from 33% to 98%.

**Conclusions:**

Musculoskeletal conditions remain a blind spot for people designing, implementing, and evaluating digital health for triage: few tools were specifically designed for musculoskeletal conditions, and most existing tools performed poorly when applied to musculoskeletal populations. We recommend health systems and clinicians use a multimodal approach, integrating both digital health tools and clinical decision-making to safely triage and diagnose until a more robust tool for musculoskeletal conditions is available. Future tool developers need to use transparent, standardized processes that prioritize tool safety, clinical value, and trustworthiness when designing for clinicians and patients.

## Introduction

Musculoskeletal conditions are one of the largest contributors to the global burden of disease and the sixth largest contributor to disability worldwide [1]. The global forecast predicts that the burden of musculoskeletal conditions will more than double in the decades between 2020 and 2050 [1]. Most musculoskeletal conditions can be effectively managed proactively in a primary care setting, not in the emergency department (ED) [2]. Our recent analysis of epidemiological data revealed that approximately 1 in 10 ED visits were related to musculoskeletal issues, and 6 in 10 of these cases could have been appropriately managed outside the ED [3]. This indicates a need to re-evaluate how people with musculoskeletal conditions access health care.

Effective and efficient triage processes are needed to help patients navigate health systems and find timely and high-quality care, avoiding inappropriate use of the ED [4]. The idea of triage was first applied in military settings to help allocate resources and timely care for the wounded [4]. In today’s context, triage is often considered in the ED or the first point of contact for clinicians to help prioritize who needs attention first when patients present to the ED [4]. In regard to triaging musculoskeletal conditions, triage is often conducted by tele-triage, paper-based triage, and face-to-face triage [5]. However, patients and musculoskeletal experts have reported that these approaches are inefficient and ineffective in moving patients through the health system [5].

There is an increasing trend toward the use of digital triage, such as online symptom checkers by patients, to make an informed decision on the next and best course of action for their current problem [6-8]. More recently, the World Health Organization has launched a global strategy on digital health to help improve the health and well-being of all humans [9]. This includes defining digital health as “the use of information and communications technology in support of health and health-related fields,” which encompasses eHealth, mobile health (mHealth), advanced computer sciences, such as big data and artificial intelligence (AI) or machine learning, and the broad scope of telehealth and telemedicine [10]. Digital health tools have the potential to tackle overcrowding in the ED and primary care settings by guiding patients to alternative services for musculoskeletal care that may be just as effective as the ED.

In the last decade, there has been a shift toward integrating digital health tools, such as symptom checkers, into the health system, as reflected in the volume of reviews evaluating such tools [6,8,11-14]. With the proliferation and widespread adoption of generative AI (eg, large language models [LLMs]), the public seems accepting of using digital health tools like AI to provide guidance and diagnoses for health conditions. This is despite generative AI not being specifically designed for health care use [15]. These findings reflect the growing demand for the integration of digital technology into health care.

Despite the advancement of digital health tools, there are no reviews currently available that have studied digital health tools for diagnosing and triaging musculoskeletal conditions [8,11-14]. Understanding the available tools, including their performance (eg, accuracy), will help researchers, policymakers, and clinicians tailor future digital health technologies for musculoskeletal conditions and make informed decisions about how to implement technology in health systems. Helping patients find the “right care at the right time” for musculoskeletal conditions may help reduce burden on the health system and allow the ED to do what it was created for: provide life-saving care.

The primary objective of this review was to identify and describe available digital health tools that can triage and diagnose musculoskeletal conditions in primary, urgent, and emergency settings. The secondary objective was to summarize the performance and accuracy of digital health tools.

## Methods

### Overview

This scoping review was conducted in accordance with the Johanna Briggs Institute methodology for scoping reviews [16,17] and reported following the PRISMA-ScR (Preferred Reporting Items for Systematic Reviews and Meta-Analyses Extension for Scoping Reviews; Checklist 1) and PRISMA-S (Preferred Reporting Items for Systematic Reviews and Meta-Analyses Extension for Search) [18,19]. We were guided by Arksey and O’Malley’s [20] framework with the additions of Levac et al [21]. A protocol was prospectively registered on the Open Science Framework (OSF). Amendments to the protocol were updated and uploaded to the OSF [22].

### Search Strategy

An electronic search was conducted in 6 databases (MEDLINE [OVID], Embase [OVID], CENTRAL [OVID], CINAHL [EBSCO], Compendex, and Web of Science) and 4 gray literature sites (OpenGrey, GoogleScholar, arXiv, and medRxiv) with the aid of a biomedical librarian and information specialist. Our search strategy was not peer-reviewed but was tested through an iterative process by the biomedical librarian to ensure that search strategies returned identified seed papers. Initial search strategies were adapted from previously published work [11,14]. We searched databases and gray literature from inception to September 18, 2025. Table 1 provides the population-concept-context framework for our search strategy and illustrates how we operationalized our search. The full MEDLINE search strategy is outlined in Multimedia Appendix 1, and all other search strategies are uploaded and available on OSF [22]. To supplement the search, we screened the reference lists of relevant reviews and included records. We also searched the Cochrane Database of Systematic Reviews, PROSPERO, OSF, and *JBI Evidence Synthesis* to identify any active systematic or scoping reviews on the topic. The search approach for identifying gray literature is detailed in Multimedia Appendix 2.

**Table 1.** Population-concept-context (P-C-C) framework.

P-C-C	Definition	Keywords
Population (people with musculoskeletal pain)	Acute (traumatic) or chronic injury related to muscles, bones, joints, tendons, or ligaments problems that cause regional or generalized pain[23] and musculoskeletal disease or conditions as defined by the Global Burden of Disease (rheumatoid arthritis, osteoarthritis, low back pain, neck pain, and gout) [1].	(Musculoskeletal or MSK) injur* or pain* or tear* or ligament* or sprain* or strain* or gout or arthritis or rheumatic arthritis
Concept (digital health)	“The use of information and communications technology in support of health and health-related fields” [10]. Digital health captures eHealth, mobile health, advanced computer sciences, such as big data and AI^a^, and the broad scope of telehealth and telemedicine [10].	telemedic* or telehealth or teletriag* or teleconsult* or telecare or tele-care or virtual medicine or virtual care or virtual triage or digital health or digital tool or digital care or digital health technology or AI or artificial intelligence or deep learning or machine learning
Context (triage)	Triage guides the distribution of medical resources to patients when there is a scarcity of health care resources and often refers to a process to allocate, ration, or prioritize patient treatment and is considered first point-of-contact care [4]. Triage can be done by a clinician, patient, or technology (eg, AI) and may involve patients’ self-assessment.	self-refer* or self-assess* or self-access* or tele-triage or triage or diagnosis or decision making or symptom checker*

a AI: artificial intelligence.

### Inclusion Criteria

Studies of adults (aged 18 years and older) with musculoskeletal conditions (≥25% of the sample had to be musculoskeletal-related) that identified and reported a digital health tool designed specifically to triage or diagnose in primary, urgent, or emergency care settings were included. Textbox 1 describes the inclusion and exclusion criteria. We excluded studies that evaluated the effectiveness of virtual assessments. We also excluded studies that used digital health tools for secondary diagnoses (ie, patient had already seen a practitioner and given a diagnosis), as the tools were typically used to manage symptoms and not for primary triage or diagnosis.

Textbox 1. **Overview of study selection criteria.**

**Inclusion criteria**

- Adult participants (≥18 years) with a primary complaint of a musculoskeletal condition
- Sample has ≥25% musculoskeletal conditions
- Identifies and reports a digital health tool designed specifically for triage or diagnosis in primary care, urgent care, or emergency settings

**Exclusion criteria**

- Not English language
- Nonhuman data (eg, vignettes or simulated clinical cases)
- Study design (not original data, eg, review, opinion paper, commentaries, and guidelines)
- Not adult population (all participants aged at least 18 years)
- Not related to a digital health tool (instrument testing or replication or validation studies of clinician assessment to virtual assessment were excluded, no wearable or technology testing was excluded)

### Study Selection

Records were collated and uploaded into EndNote (version 20.3; Clarivate Analytics), and duplicates were removed before uploading to Covidence (Veritas Health Innovation) for screening. Pairs of independent reviewers screened all records by title and abstracts, and a third reviewer (CLA) resolved any discrepancies if consensus could not be reached.

At the full-text stage, we first conducted pilot screening, where all reviewers assessed the same 5 full texts. If major discrepancies were identified, we met to review and discuss how to apply the screening criteria in a standardized manner. All full-text papers were reviewed by pairs of independent reviewers. Reasons for exclusion during full-text screening were recorded. Any disagreements between the reviewers at each stage of the selection process were resolved through consensus or by an additional reviewer (CLA) as required.

### Data Extraction

Data were extracted from included records independently by pairs of reviewers using a custom data extraction tool designed in Microsoft Excel by the research team. Any disagreements were resolved via consensus. We extracted the following details where available: study characteristics (author, country, sample size, and study aim), participants’ demographics (sex, age, and musculoskeletal pain or diagnosis), type of digital tool (name, purpose of tool, and target users), design and development process, platform of tool, tool delivery (eg, clinician or patient self-access), context or care setting in which the tool was used, assessment of performance or accuracy results, and key findings relevant to the review question. Where relevant, authors were contacted once via email to request missing data and to clarify details about the digital health tool.

### Data Synthesis

Studies were summarized by study characteristics, digital health tool details, and performance assessment. Descriptive data were summarized as proportions when appropriate. Digital health tools were classified according to the World Health Organization digital health category [10] (eHealth, mHealth, and AI or machine learning), function (ie, triage, diagnosis, or both), care setting in which the tool was used (ie, ED, primary or urgent care, or mixed), research setting (ie, urban, rural, or both), how the tool was administered (eg, self-access or clinician-delivered), technology interface (eg, web-based or app), and intended user (patient-facing, clinician-facing, or both).

Digital tools were rated using the technology readiness level (TRL) and associated technology stage by the first author (LKT) and verified by a second rater [24]. The TRL ranges from 1 to 9, with 1 representing tool conception and 9 representing that the tool is ready to be used in real-world settings [24]. We classified TRL across technology stages (fundamental research, research and development, pilot and demonstration, early adoption, and commercially available) [24].

When available, the performance of the digital health tool was reported by identifying appropriate triage referrals or recommendations or diagnoses compared to a reference standard (eg, physician diagnosis). Measures of performance included diagnostic test accuracy (area under the receiver operator characteristic curve, sensitivity, specificity, positive predictive value, negative predictive value, and likelihood ratio) or reliability measures (internal consistency, test-retest reliability, and intra- or interrater reliability).

### Deviations From Protocol

We sought to use the continuous active learning on Covidence to support title and abstract screening [25]. During attempts to calibrate the algorithm, the algorithm did not perform well in identifying relevant papers for this review. We were not confident in screening titles and abstracts with only 1 human reviewer (LKT). Instead, all titles and abstracts and full text records were screened in duplicate by 2 independent human reviewers.

As scoping reviews are an iterative process and aim to assess and evaluate the available evidence, a broad research question often results in a highly sensitive search and less specific records. We made pragmatic decisions and minor amendments to the selection criteria as the review progressed. At the full-text screening stage, studies including a general population had to report ≥25% of the sample being musculoskeletal-related to be included. This cutoff was determined based on studies that indicated the prevalence of musculoskeletal presentations in ED was approximately 25% [26,27].

## Results

### Overview

**Figure 1.**
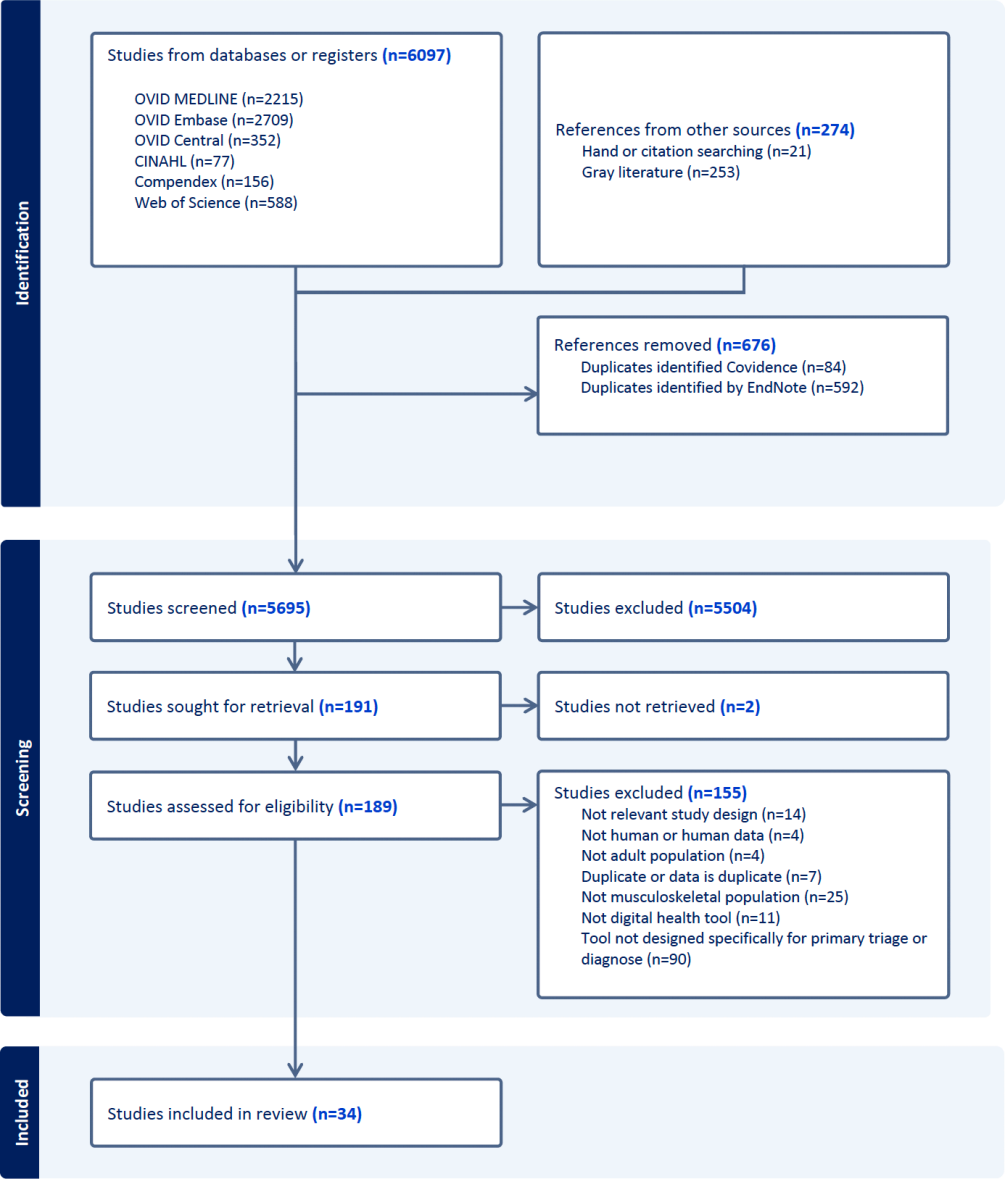
PRISMA-ScR (Preferred Reporting Items for Systematic Reviews and Meta-Analyses Extension for Scoping Reviews) flowchart.

**Figure 2.**
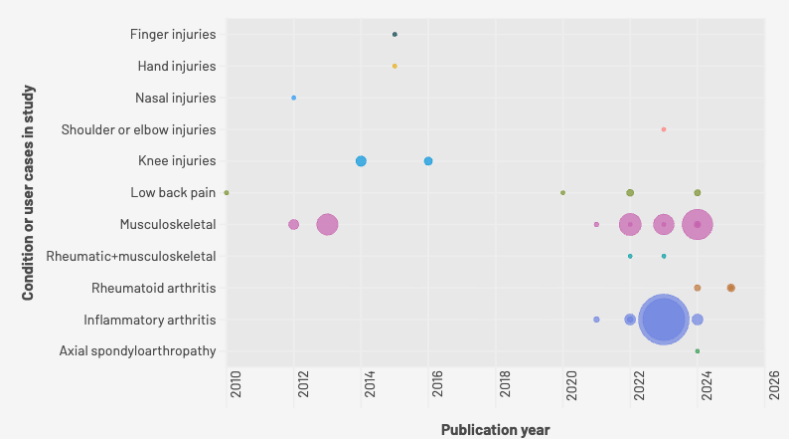
Sources of data charted by publication year and condition.

The titles and abstracts of 5695 unique records were screened, and 189 papers were reviewed in full (Figure 1). In total, 34 studies met the inclusion criteria (n=37,509 participants across 33 studies, 12,470/37,509, 33% female). The median age was 50 (range 18-91) years. One study did not report the sample size. Sex and age data distribution were missing in 12 and 13 studies, respectively. A list of the studies that were excluded at the full-text stage is presented in Multimedia Appendix 3. Figure 2 charts the data of all 34 studies included by condition, publication year, and sample size of the study, if reported.

### Study Characteristics

In total, 30 of 34 (88%) studies focused on primarily musculoskeletal conditions, and 4 of 34 (12%) studies focused on general populations with a subset of the sample being musculoskeletal conditions. Full study details can be found in Multimedia Appendix 4 [28-61].

In total, 25 of 34 (74%) studies were peer-reviewed, 7 of 34 (21%) records were conference abstracts, and 2 of 34 (6%) were industry case reports. Studies were published between 2010 and 2025. Cross-sectional (10/34, 29%) studies were most common, followed by nonrandomized or quasi-experimental studies (8/34, 24%), randomized controlled trials (5/34, 15%), retrospective cohort (4/34, 12%), mixed or multimethods (3/34), prospective observational cohort (2/34), and case report (2/34, 6%). All studies were conducted in high-income countries (n=1 country not reported), with the majority being from Europe (21/34, 62%) or the United States (7/34, 21%).

Digital health tools were evaluated across various care settings, including ED or urgent care (6/34, 18%), physician-led primary care (6/34, 18%), physiotherapist-led primary care (1/34, 3%), patient self-access (19/34, 56%), and mixed (eg, primary care and ED) settings (2/34, 6%).

### Tool Identification and Characteristics

#### Overview

Inflammatory arthritis (eg, rheumatoid arthritis, gout, and spondyloarthropathy) and arthritis-related conditions were the most common (13/34, 38%) musculoskeletal conditions studied, followed by generic musculoskeletal conditions (11/34, 32%). Others tested digital tools for low back pain (4/34, 12%), knee (2/34, 6%), finger or hand (2/34, 6%), shoulder (1/34, 3%) conditions, and nasal fractures (1/34, 3%).

We identified 16 unique digital health tools (Table 2). In total, 7 studies did not report the name of the digital health tool that was studied or studied a bespoke tool designed for the study where we could not extract the name of the tool. Of the 34 tools, 13 (38%) tools reported that they were designed to “diagnose and triage,” 12 (35%) tools were designed to diagnose, and 9 (26%) tools were designed to triage (Table 2). Only 2 tools (Phio [32,39] and Digital Assessment Routing Tool [DART] [51,52]) were designed specifically to triage musculoskeletal conditions. Overall, 3 tools (ADA [37,41,46-49], Buoy Health [33], and WebMD Symptom Checker [40]) were generic health tools that had integrated algorithms to screen musculoskeletal conditions (among other conditions). One tool used OpenAI or LLMs (ChatGPT [29,34,49]) and reported algorithms capable of diagnosing musculoskeletal conditions. Five tools (Rheumatic? [43,45,53,56], Rheport [46-48], RheumConnect [60], ReumAI [38], and Bechterew-check [41]) were condition-specific (ie, designed for rheumatological or inflammatory conditions) with capabilities to differentiate these conditions from other types of musculoskeletal conditions. Two tools were joint-specific (Therapha for low back pain [28] and Virtual Knee Doc for acute knee injuries [30,31]).

**Table 2.** Characteristics of digital health tools, summarizing purpose, use, technology development, and availability.

Digital health tool name	Purpose of tool^a^	Type of tool^b^^,^ intended user, and access level	Tool format	Digital health category	Technology readiness level^c^	Technology readiness assessment^d^	Tool processes^e^	Available to public
ADA [37,41,46-49]	Diagnosis and triage	Symptom checker (patient; self-access)	App	mHealth^f^, AI^g^ or ML^h^	9	Commercially available	AI	Yes
Bechterew-check [41]	Diagnosis	Symptom checker (patient; self-access)	Web-based	mHealth	7	Pilot and demonstration	Clinical or decision support pathway	Yes (only in German)
Buoy Health [33]	Diagnosis and triage	Symptom checker (patient; self-access)	Web-based or app	eHealth, mHealth, AI or ML	9	Commercially available	AI	Yes
ChatGPT [29,34,49]	Diagnosis	Diagnostic predictor (patient; self-access)	Web-based	eHealth, AI or ML	9	Commercially available	AI	Yes
Digital Assessment Routing Tool [51,52]	Triage	Digital triage^i^ (patient; self-access)	App	mHealth	8	Research and development	Clinical or decision support pathway	No
Phio [32,39]	Diagnosis and triage	Symptom checker^i^ (patient; self-access)	App	mHealth, AI or ML	9	Commercially available	AI	No, Proprietary
Phone camera [42] (any built-in camera)	Triage	Tele-triage (clinician; clinician-administered)	Phone	mHealth	6	Pilot and demonstration	Clinical or decision support pathway	Yes
PhysioDirect [44]	Diagnosis and triage	Tele-triage (clinician; clinician-administered)	Phone	eHealth	6	Pilot and demonstration	Clinical or decision support pathway	No
Rheumatic? [43,45,53,56]	Diagnosis and triage	Symptom checker (patient; self-access)	Web-based	eHealth	8	Pilot and demonstration	Clinical or decision support pathway	Yes
Rheport[ [37,46-48]	Diagnosis	Symptom checker (patient; self-access)	Web-based	eHealth	9	Early adoption	Clinical or decision support pathway	Yes (only in German)
Therapha [28]	Diagnosis and triage	Digital triage (clinician; clinician-administered)	Web-based	eHealth, AI or ML	9	Commercially available	Clinical or decision support pathway	No (propriety)
TriageXpert Dual Purpose [50]	Triage	Tele-triage (clinician; clinician-administered)	Phone	eHealth	7	Commercially available	Clinical or decision support pathway	No
RheumConnect [60]	Triage	Symptom checker (patient; self-access)	Web-based chatbot	eHealth, AI or ML	6	Pilot and demonstration	AI	No
ReumAI [38]	Triage	Tele-triage (clinician; clinician-administered)	Phone	eHealth, AI or ML	6	Pilot and demonstration	AI	No
Virtual Knee Doc [30,31]	Diagnosis	Symptom checker (patient; self-access)	Web-based	eHealth	6	Pilot and demonstration	Clinical or decision support pathway	No
WebMD Symptom Checker [40]	Diagnosis	Symptom checker (patient; self-access)	Web-based	eHealth, AI or ML	9	Commercially available	AI	Yes

a Triage: provide next steps for care based on symptoms and urgency, may provide preliminary diagnoses but not the objective of the tool. Diagnosis: provide a preliminary diagnosis based on symptoms, which aids to direct next steps in care.

b Type of tool: symptom checker: tool designed for patients to enter their symptom data; tele-triage: tool designed to triage using telephone interface; digital triage: tool designed to triage using eHealth or mHealth interface; diagnostic predictor: tool designed to use data to predict diagnosis or triage pathway.

c Based on Innovation Canada Technology Readiness Level: rated on a scale of 1-9, where 1=tool conception and 9=tool ready for real-world settings.

d Based on Innovation Canada Technology Readiness Stages: fundamental research, research and development, pilot and demonstration, early adoption, commercially available.

e Tool processes: AI: tool that uses big data to assign probability to allow for computer-driven decision-making; clinical decision support pathway: predefined decision tree or rule-based algorithms that support clinical decision-making.

f mHealth: mobile health.

g AI: artificial intelligence.

h ML: machine learning.

i Self-access within the UK National Health Service.

#### Intended Users

Most studies (24/34, 71%) reported on digital health tools designed for use by patients, 10 (10/34, 29%) studies targeted tools at clinicians. In total, 19 (19/34, 56%) studies reported on tools that were symptom checkers and were designed to be patient-facing. A total of 10 (29%) studies reported on clinician-facing tools for triage, diagnosis, or diagnostic prediction.

#### Patient-Facing

We classified ADA, Buoy Health, ChatGPT, Bechterew-check, Rheport, Rheumatic?, RheumConnect, Virtual Knee Doc, DART, and Phio as tools for patients. All used an app or a web-based interface. ADA, Buoy Health, and ChatGPT were tools used for generic health purposes, while the others were designed for specific groups of conditions (ie, rheumatological or musculoskeletal conditions). DART and Phio were designed to integrate with the UK National Health Service. Patients who used DART or Phio had their results forwarded to a primary care team or physiotherapist.

#### Clinician-Facing

In total, 7 tools were identified for clinicians. Therepha is a clinical decision support system designed for physiotherapists to diagnose and triage low back pain and was piloted in the ED [28]. ReumAI uses tele-triage where a nonphysician staff uses AI-guided telephone interviews to identify diagnoses and potential previsit tests [38]. Triage Xpert Dual Purpose [50] and PhysioDirect [58] were triage tools designed for implementation within specific health systems. One study examined triage of nasal fractures using a built-in camera to triage to the right hospital setting [42], and another used tele-triage to assess whether the ED could be avoided altogether for finger injuries [36]. Most clinician-facing triage tools used tele-triage (ie, phone call) as their interface, except for Therepha, which was a web-based tool. In total, 2 studies leveraged large datasets and AI to predict diagnoses, with 1 study using ChatGPT [29,60].

### Performance and Usability

Of the 34 studies identified, 19 (56%) evaluated the performance of the digital health tool (Table 3). The most common definition and method to determine performance was measuring concordance to a clinician diagnosis or recommended triage pathway, often by evaluating sensitivity and specificity. The performance of these tools varied widely and was partly dependent on the context in which they were being used (eg, ED or primary care). Sensitivity ranged from 39% to 91%, and specificity from 23% to 80% [28,30,31,37,41,45,46,48]. The methods for measuring accuracy were poorly reported, often in the form of proportion of correct triage or diagnoses. Reported accuracy ranged from 33% to 98% across 12 unique tools (n=16 studies) [28,29,34,36-38,41,45,49,51,59,61]. The accuracy of tools used by patients in tertiary settings (eg, seeking care from a specialist such as an orthopedic surgeon or rheumatologist) was reported as higher than the accuracy of tools used in primary care settings.

For studies that compared digital health tools against each other, ADA was the most common tool used for comparison. When compared to rheumatologists and medical students, ADA was superior to clinician’s diagnosis of rheumatic and nonrheumatic conditions, ChatGPT, and Bechterew-check [37,41,49]. ADA was comparable to Rheport for diagnostic accuracy of rheumatic conditions [48]. ChatGPT performed similar to experienced rheumatologists for potential diagnostic accuracy for rheumatic conditions [49].

In total, 4 tools were available in multiple languages (ADA, ChatGPT, Rheumatic?, and WebMD Symptom Checker), and 8 tools were accessible to the public; however, 2 were designed for German speakers (Table 3). Based on the TRL, we classified 6 tools as being at the commercially available stage (ADA, Buoy Health, ChatGPT, Phio, Therapha, and WebMD Symptom Checker).

**Figure 3.**
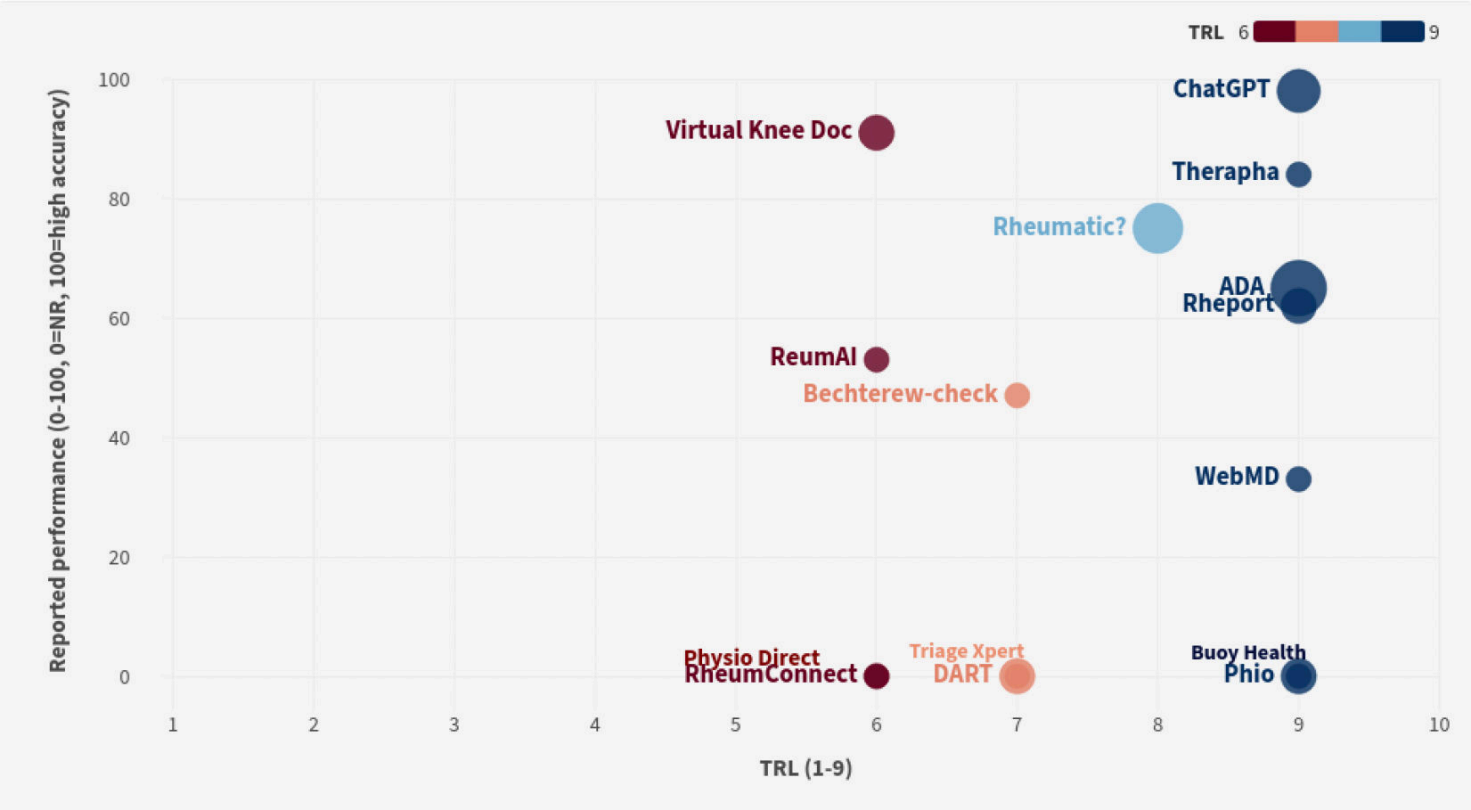
Visualization comparing TRL and the highest reported performance evaluation across identified digital health tools. NR: not reported; TRL: technology readiness level.

Figure 3 provides a visualization comparing TRL and performance evaluation for the identified digital health tools (Only 15 of the 16 identified digital tools are reported in this figure. The “phone built in camera” was not graphed.). If the tool did not complete a performance evaluation of the tool, a 0 was given for reported performance (ie, accuracy) on Figure 3. Despite some tools being commercially available, there was a discrepancy in reported performance findings for musculoskeletal conditions.

**Table 3.** Performance statistics of digital health tools.

Digital health tool and authors		Performance of tool evaluated (yes or no)	Definition used to define tool performance	Condition evaluated	Methods to evaluate performance	Sensitivity (%)	Specificity (%)	Accuracy of tool^a^	Other findings reported (%) (95% CI)
ADA^b^									
	Knitza et al (2021) [46]	Yes	Concordance with physician diagnosis	Rheumatic	Sensitivity or specificity, PPV^c^, NPV^d^	43	64	NR^e^	PPV 37 (26-48), NPV 69 (60-80)
	Knitza et al (2024) [48]	Yes	Concordance with physician diagnosis	Rheumatic	Sensitivity or specificity, PPV, NPV	52	68	NR	PPV or NPV varied depending on whether ADA or Rheport was used first
	Graf et al (2022) [37]	Yes	Concordance with identified diagnosis from clinical trial	Rheumatic	Sensitivity or specificity, accuracy	71	64	54% accurately diagnosed same condition	NR
	Hannah et al (2024) [41]	Yes	Concordance with discharge summary report	Rheumatic	Sensitivity or specificity, accuracy	39	78	58% accurately diagnosed same condition	NR
	Krusche et al (2024) [49]	Yes	Concordance with physician diagnosis	Rheumatic	Proportion	NR	NR	65% accurate for all cases; 71% accurate for cases with IRDs^f^; 61% accurate for non-IRD cases	NR
Bechterew-check									
	Hannah et al (2024) [41]	Yes	Concordance with discharge summary report	Axial spondyloarthropathy	Sensitivity or specificity, accuracy	41	53	47% accurately diagnosed same condition	NR
Bespoke tool (no name reported)									
	Demmelmaier et al (2010) [35]	No	NT^g^	Low back pain	NT	NT	NT	NT	NR
	Martin and Payne (2020) [54]	No	NT	Low back pain	NT	NT	NT	NT	NR
	Phillips et al (2012) [55]	No	NT	MSK^h^	NT	NT	NT	NT	NR
	Ryan and Grinbergs (2024) [57]	No	NT	MSK	NT	NT	NT	NT	NR
	Trivedi et al (2024) [61]	Yes	Concordance with nurse triage	MSK	Proportion	NR	NR	63% accurately triage	NR
	Soin et al (2022) [59]	Yes	Concordance with physician diagnosis	Low back pain	NR	NR	NR	72% software predicted correct diagnosis	NR
Buoy Health									
	Carmona et al (2022) [33]	No	NT	Generic MSK	NT	NT	NT	NT	NR
ChatGPT									
	Badsha et al (2024) [29]	Yes	Concordance with physician diagnosis	Rheumatic	NR	NR	NR	98% accurate with rheumatologist diagnosis	NR
	Daher et al (2023) [34]	Yes	Concordance with physician diagnosis	Shoulder or elbow injuries	NR	NR	NR	93% accurate with surgeon diagnosis; 83% accurate with surgeon management	NR
	Krusche et al (2024) [49]	Yes	Concordance with physician diagnosis	Rheumatic	Proportion	NR	NR	35% accurate for all cases; 71% accurate for cases with IRDs; 15% accurate for non-IRD cases	NR
Digital Assessment Routing Tool (DART)									
	Lowe et al (2022) [51]	Yes	Concordance with physiotherapist expert	MSK	Proportion	NR	NR	84% DART matched physiotherapist	NR
	Lowe et al (2024) [52]	Yes	Concordance with physiotherapist expert	MSK	Intraclass correlation coefficient (ICC)	NR	NR	NR	ICC 0.37 (0.16‐0.55)
Phio									
	Bond et al (2024) [32]	No	NT	NT	NT	NT	NT	NT	NR
	Gymer et al (2023) [39]	No	NT	NT	NT	NT	NT	NT	NR
Phone Camera									
	Hara et al (2015) [42]	Yes	Accuracy of triage recommendations	Finger injuries	NR	NR	NR	NR	NR
PhysioDirect									
	Kelly et al (2021) [44]	No	NT	NT	NT	NT	NT	NT	NR
Rheport									
	Knitza et al (2021) [46]	Yes	Concordance with physician diagnosis	Rheumatic	Sensitivity or specificity, PPV, NPV	54	52	NR	PPV 35 (25-47); NPV 70 (58-79)
	Knitza et al (2024) [48]	Yes	Concordance with physician diagnosis	Rheumatic	Sensitivity or specificity, PPV, NPV	62	47	NR	PPV or NPV varied depending on whether ADA or Rheport was used first
Rheumatic?^i^									
	Knevel et al (2022) [45]	Yes	Concordance with physician diagnosis/treatment recommendation	Rheumatic	Sensitivity or specificity, AUC-ROC^j^	67	72	AUC-ROC 75 (95% CI 62‐89)	NR
	Qin et al (2024) [56]	No	NT	Rheumatic	NT	NT	NT	NT	NR
	Lundberg et al (2023) [53]	No	NT	Rheumatic	NT	NT	NT	NT	NR
	Jakobi et al (2025) [43]	No	NT	Rheumatic	NT	NT	NT	NT	NR
ReumAI									
	Gómez-Centeno et al (2025) [38]	Yes	Concordance with physician diagnosis	Rheumatic	NR	NR	NR	53% accurate with rheumatologists	NR
RheumConnect									
	Tan et al (2023) [60]	No	NT	Rheumatic	NT	NT	NT	NT	NT
Therapha									
	Badahman et al (2024) [28]	Yes	Concordance with MRI findings	Low back pain	Sensitivity or specificity, PPV, NPV, ROC^k^	88	80	ROC 0.84 (95% CI 0.6‐1.0; *P*=.001)	PPV 99 (25-47); NPV 27 (58-79)
Triage Xpert Dual Purpose									
	Li et al (2023) [50]	No	NT	MSK	NT	NT	NT	NT	NT
Virtual Knee Doc									
	Bisson et al (2014) [30]	Yes	Concordance with physician diagnosis	Knee injuries	Sensitivity or specificity	89	27	NR	NT
	Bisson et al (2016) [31]	Yes	Concordance with physician diagnosis	Knee injuries	Sensitivity or specificity	91	23	NR	NT
WebMD Symptom Checker									
	Hageman et al (2015) [40]	Yes	Concordance with physician diagnosis	Hand injuries	Proportion	NR	NR	33% accurate with hand surgeon diagnosis	NT

a Reported values from the study and not interpretation of authors.

b Findings reported from ADA diagnosis 1 (D1) in study.

c PPV: positive predictive value.

d NPV: negative predictive value.

e NR: not reported.

f IRD: inflammatory rheumatic disease.

g NT: not tested.

h MSK: musculoskeletal.

i Findings reported from dataset A in study.

j AUC-ROC: area under the receiver operating curve.

k ROC: receiver operating curve.

## Discussion

### Principal Findings

We aimed to identify and describe the available tools for triaging and diagnosing musculoskeletal conditions in primary, urgent, and emergency settings. Based on a synthesis of 34 studies and data from 16 different digital health tools, there were no digital health tools with sufficient evidence to support effective triage and diagnosis of musculoskeletal conditions across these settings. Approximately half of these tools were available to the public. Not all tools were available in English, with 2 tools only available in German (Bechterew-check and Rheport). The most frequently studied digital health tool was ADA (n=5), followed by Rheumatic? (n=4), then ChatGPT (n=3). Only 2 tools (DART and Phio) were purposely developed for screening musculoskeletal conditions. Both tools are not currently available outside of the UK’s National Health Service. We were surprised to find so few digital health tools targeting musculoskeletal conditions, given the substantial global burden of musculoskeletal conditions [1]. Notably, rheumatological or inflammatory arthritis was the most prevalent musculoskeletal condition studied, despite low back pain being the most common musculoskeletal condition seen in ED and primary care settings [62]. We identified 4 studies that included digital health tools targeting low back pain, but only 1 of these reported which tool was used (Therapha). Our findings reflect the discordance of research across digital health technology and the current health landscape. Many tools were inaccessible or not designed for practical use in managing musculoskeletal pain, the most burdensome conditions seen in primary care.

Our secondary objective was to summarize the performance and accuracy of the included digital health tools. Approximately 50% of the studies evaluated the performance of a digital health tool. Apart from ChatGPT, most generic digital health tools (eg, ADA and WebMD Symptom Checker) reported poor accuracy (often less than 50% accuracy in identifying the correct diagnosis compared to clinicians) for musculoskeletal conditions [37,40,48]. Despite the use of ChatGPT by the public as a symptom checker [15], ChatGPT’s accuracy for diagnosing musculoskeletal and rheumatic conditions was variable, ranging from 33% to 98% [29,34,49]. We suggest that further research is needed before considering ChatGPT as an accurate diagnostic or screening tool. Tools that were designed to diagnose peripheral or spinal musculoskeletal conditions (eg, low back pain or knee injuries) appear to be more promising with high sensitivity (88%‐91%) [28,31]. Finally, tools designed specifically to triage (rather than diagnose) musculoskeletal conditions (ie, Phio and DART) demonstrated the best performance. Recent findings published on DART and Phio indicate that these tools have high agreement (>90%) with expert physiotherapist recommendations on next care pathways [63,64]. However, the heterogeneity across evaluation methods highlights the importance of standardized development and evaluation frameworks to ensure that digital triage tools for musculoskeletal conditions are accurate, transparent, and safe before integrated into clinical settings and workflows.

### Not Yet Ready for Prime Time

One of the key findings of our review is that some tools are commercially available and integrated into health systems for musculoskeletal screening without robust methodological evaluation or reporting. Premature implementation raises concerns, particularly given the risk of misdirecting patients or delaying appropriate care. Before being adopted at scale, digital triage tools must demonstrate value in real-world settings and meet minimum standards for safety, accuracy, and usability. However, many studies evaluating these tools lack transparent reporting, making it difficult to assess how performance claims were derived. Studies reporting high accuracy of their digital tools often had poor transparency or a lack of details on their tool evaluation. We suggest caution with interpreting these digital health tools as ready for public use without further evaluation. It is also unclear how tools that use LLMs operate. These networks are often termed “black boxes” due to the inability to explain how these systems achieve their output [65].

Our findings have been confirmed by a recent study that evaluated diagnostic accuracy and clinical reasoning using 6 different generative AI (LLMs) for rheumatic diagnoses [66]. Despite the LLMs reporting high diagnostic accuracy (~80%), all models reported subpar clinical reasoning quality (eg, explaining reasons for supporting diagnoses) [66]. These findings underscore the importance of digital health tools requiring both high diagnostic accuracy alongside transparent algorithms to help to explain the logic behind the tool’s decision. To improve transparency and enable reproducibility, it is important to establish standards for incorporating ethical AI in digital health. Without transparency in how tools were developed, or in the algorithms used, it is unclear whether the tools are safe for the public to use.

The only digital health tool with robust evaluation of its performance was the generic health app, ADA, which is a Conformité Européenne–certified medical product [46,48]. ADA’s performance was inconsistent across the studies, and ADA correctly identified the musculoskeletal condition or triage option in fewer than half of the cases [37,41,46,48,49]. Condition-specific digital health tools (Rheport [46,48], Rheumatic? [45], Virtual Knee Doc [30,31], Therapha [28], and ReumAI [38]) performed slightly better. The reported accuracy was higher in these tools, especially if these tools were implemented in tertiary care settings (outside of the ED or primary care). We are not aware of an acceptable threshold for performance (ie, accuracy) for digital health tools. However, we recommend implementing tools that are at least more accurate than flipping a coin and provide consistent results across different study contexts or musculoskeletal conditions.

AI-driven tools, like ADA or ChatGPT, may perform better than clinician decision support systems or physicians or rheumatologists in diagnosing rheumatic conditions [49,67]. Integrating digital health tools in tandem with other nonspecialist professions (eg, general practitioners and allied health professionals) could help guide patients to their next care steps as they wait for specialists (eg, rheumatologists) or avoid unnecessary visits to specialists or other care providers. AI-driven tools that have included diagnostic findings (eg, imaging, clinical symptoms or signs, and bloodwork) have superior diagnostic accuracy to other AI models [41,67,68]. Until robust stand-alone digital health tools are developed (ie, a symptom checker that can be used independently by patients), combining digital health tools and clinician feedback may be the best method to streamline diagnosis and care in complex cases while providing timely care for common musculoskeletal conditions.

Several frameworks for evaluating digital health tools have been proposed [69]. A recent scoping review identified 12 key domains—ranging from tool description and content to safety, clinical effectiveness, and efficacy—across 95 frameworks that developers and researchers can draw on [69]. However, the heterogeneity reflects a broader challenge: many digital health tools span multiple categories (eg, eHealth or mHealth tools incorporating AI), making classification inconsistent and evaluation difficult. Advancing this field requires standardized terminology, harmonized testing and evaluation frameworks, and clear reporting guidelines—crucial steps to ensure both progress and patient safety.

### Why Would a Digital Health Tool Do a Poor Job at Screening Musculoskeletal Conditions?

Through the process of screening studies for inclusion into our review, we found definitions of musculoskeletal conditions that were vague and varied widely. Definitions of “musculoskeletal” are often limited to orthopedic conditions or pain related to musculoskeletal structures [1,23]. However, musculoskeletal conditions are a complex category involving heterogeneous conditions, such as rheumatological or inflammatory arthritis or gout, that are not typically grouped as musculoskeletal in clinical practice. We relied on a broad definition to capture specific musculoskeletal conditions (eg, rheumatological conditions, arthritis, and gout) and pain related to musculoskeletal structures (eg, sprains and strains).

There is nuance in how triage would be conducted for acute versus chronic musculoskeletal conditions, including screening questions related to condition pathophysiology, subjective history, pattern of symptoms, and disability (eg, red flags), which might explain some of the variability in performance metrics of different digital tools [70]. Early diagnosis and treatment planning is often iterative for those with musculoskeletal conditions and varies depending on the condition. For example, targeted medication plays a vital role in managing rheumatological conditions [71], whereas some orthopedic conditions are managed with exercise and minimal pharmacological interventions [2]. This complexity will impact triage algorithms by influencing treatment recommendations (eg, who the patient should see) and timing of care (eg, urgent or wait-and-see). Therefore, tools that have high accuracy (ie, good performance) for triaging and diagnosing general health conditions may not necessarily have the same effectiveness when applied to musculoskeletal conditions.

Digital health tools may perform poorly at screening because of user error relating to symptom data entry and patient interaction with the tool. One solution to this is adding more key information (eg, diagnostic tests) to an AI-driven model to improve the diagnostic accuracy of the model [67]. We also suggest future work to involve patient end users to develop and refine digital health tools. Most digital health tool algorithms are derived from clinicians’ clinical reasoning, which may not follow the same thought process as a patient. In a recent qualitative study exploring how patients should be engaged in AI application to health care, patients felt that the priorities of researchers, particularly for AI tools, were to improve efficiency and effectiveness of care [72]. In contrast, patients were more interested in using AI to address issues related to accessing health care [72]. Patients should be involved early in the design and development phases to enhance the usability and understandability of digital health tools. However, patient perspectives are often included only after the digital health tool is designed. We argue that engaging patients early in the development process, such as developing the AI algorithms, may yield more acceptable and usable digital health tools.

It is unlikely that a “one size fits all” digital health tool can effectively diagnose and triage all musculoskeletal conditions. Most patient-facing tools in our review were web- or app-based tools in the form of generic symptom checkers. ChatGPT has an accessible interface and is relatively easy to use [15]. Clinician-facing tools may benefit from greater complexity or condition specificity, depending on the context in which the tools will be implemented. Instead of an either-or—general or condition-specific—we advocate for designers to consider their design goal (ie, triage or diagnosis) and intended user (ie, patient or clinician), which may improve accuracy in digital health tools for musculoskeletal conditions.

### Move (Relatively) Fast, and Try Not to Break Things

The field of digital health is growing and changing rapidly. Many health systems have been forced to move toward implementing digital health, particularly AI-driven tools, without being afforded adequate time and resources to consider safety, effectiveness, or downstream consequences [13]. This may be in part due to social and political imperatives to set key performance (productivity) indicators, transition of health care services, and drive toward greater and faster innovation. We suggest that such a climate could be dangerous for health care, especially if digital health implementation continues without adequate evidence, as our findings highlight.

There is a place for digital health triage tools used by patients and clinicians in the current health care context. Self-referral and symptom checkers can be effective for musculoskeletal conditions and to support patients’ access to care, particularly when patients do not have a consistent primary care team or provider [11]. Acute care clinics using a self-referral form found that patients with musculoskeletal conditions were accurate at self-referring, used less health care, and incurred fewer costs [73]. Emerging evidence also indicates that patients are using LLMs such as ChatGPT to make health care decisions, and it appears that the general public is accepting of using AI for health care advice and psychological support [15]. However, more research is needed to ensure that patients presenting with musculoskeletal conditions have a safe, accurate, and well-designed tool to direct them to the best care for their situation. Digital health tools also need to be designed to suit diverse populations, including those with low health literacy and limited digital literacy.

### Future Considerations and Clinical Implications

While there is a breadth of studies available for digital health and digital triage, we identified the following knowledge gaps: (1) reporting and transparency on digital health tool development must improve, (2) evaluating digital health tools needs a standard approach, (3) studying the accuracy of triage recommendations requires robust prospective studies, and (4) implementing musculoskeletal-focused digital health tools for first point-of-contact care requires attention.

Despite the absence of digital health tools for triage of musculoskeletal conditions, we are aware of other tools in development, such as SupportPrim [74], which might fill some of the knowledge gaps for health care providers. Our findings do not provide conclusive evidence to support using digital health tools to accurately screen musculoskeletal conditions in many health settings. We recommend that clinicians use these digital health tools as an adjunct to help guide patients, particularly when used as a symptom checker, but to still defer to sound clinical judgment and help patients understand the limitations of the tools.

### Limitations

Although we used a thorough search of published and unpublished data, it is possible that we have missed relevant digital health tools or papers. We set a sample threshold of at least 25% of the sample population with musculoskeletal conditions, and this may have resulted in us missing some studies (eg, studies that were just below the threshold were excluded). The threshold was intended to maximize external validity [26,27]. Our goal was to identify tools that were primarily designed to triage or diagnose (vs manage) musculoskeletal conditions. Therefore, we excluded studies and tools that were designed for self-management, even if they included a symptom checker. This led us to exclude studies that used tools for secondary triage or diagnosis (ie, used by patients who had a diagnosis or had already been seen in a primary or emergency setting) as we wanted to capture tools that could be used at the first point-of-contact. We identified some potential musculoskeletal-specific digital health tools that could be used for secondary triage or diagnosis (Multimedia Appendix 5). While we attempted to report on the performance and accuracy of the tools identified, some tools pooled data from the entire population (ie, not musculoskeletal only). Therefore, the findings may under- or overestimate the accuracy of the tool for musculoskeletal conditions. This again points to the need to design musculoskeletal-specific tools and carefully evaluate their performance.

### Conclusions

The rapid growth of AI and digital health solutions is transforming health care systems worldwide, with increasing interest in automating triage and diagnosis. However, our review shows that musculoskeletal conditions remain a blind spot: few tools were specifically designed for this purpose, and most performed poorly when applied to musculoskeletal populations. Despite commercial availability and implementation in some settings, the evidence base was weak, and tool performance was inconsistent and opaque. Health systems and clinicians should exercise caution before integrating these tools into care pathways. Musculoskeletal-specific digital tools developed through transparent, standardized processes are urgently needed to ensure safety, clinical value, and trustworthiness.

## Supplementary material

10.2196/81578 Multimedia Appendix 1 OVID MEDLINE full search strategy.

10.2196/81578 Multimedia Appendix 2 Gray literature search strategy and results.

10.2196/81578 Multimedia Appendix 3 Studies excluded at full-text stage.

10.2196/81578 Multimedia Appendix 4 Characteristics of included studies, summarizing design, demographics, and digital tool features.

10.2196/81578 Multimedia Appendix 5 Digital health tools for secondary triage or diagnosis.

10.2196/81578 Checklist 1 PRISMA-ScR checklist.

## Abbreviations

AI: artificial intelligence
DART: Digital Assessment Routing Tool
ED: emergency department
LLM: large language model
mHealth: mobile health
OSF: Open Science Framework
PRISMA-S: Preferred Reporting Items for Systematic Reviews and Meta-Analyses Extension for Search
PRISMA-ScR: Preferred Reporting Items for Systematic Reviews and Meta-Analyses Extension for Scoping Reviews
TRL: technology readiness level
