# Correction: Evidence for Digital Health Tools Designed to Support the Triage of Musculoskeletal Conditions in Primary, Urgent, and Emergency Care Settings: Scoping Review

**DOI:** 10.2196/92722

**Published:** 2026-02-26

**Authors:** Linda K Truong, James G Wrightson, Raphaël Vincent, Eunice Lui, Jamon L Couch, Ellen Wang, Cobie Starcevich, Dean Giustini, Alex Haagaard, Elena Lopatina, Niels van Berkel, Michael Skovdal Rathleff, Clare L Ardern

**Affiliations:** 1 Centre for Aging SMART, University of British Columbia, Vancouver, BC, Canada; 2 Department of Physical Therapy, Faculty of Medicine, University of British Columbia, 13737 96 Avenue, Surrey, BC, V3V 0C6, Canada, 1 604 822 4519; 3 Department of Family Practice, Faculty of Medicine, University of British Columbia, Vancouver, BC, Canada; 4 School of Rehabilitation, Faculty of Medicine, Université de Montréal, Montreal, QC, Canada; 5 Hôpital Maisonneuve-Rosemont Research Center, Université de Montréal Affiliated Research Center, Montreal, QC, Canada; 6 Centre for Interdisciplinary Research in Rehabilitation of Greater Montreal, Institut Universitaire sur la Réadaptation en Déficience Physique de Montréal, Montreal, QC, Canada; 7 La Trobe Sport and Exercise Medicine Research Centre, La Trobe University, Melbourne, Australia; 8 Arthritis Research Canada, Vancouver, BC, Canada; 9 School of Allied Health, Faculty of Health Sciences, Curtin University, Perth, Australia; 10 Physiotherapy Department, Rockingham General Hospital, South Metropolitan Health Service, Perth, Australia; 11 Biomedical Branch Library, University of British Columbia, Vancouver, BC, Canada; 12 Pain BC, Vancouver, BC, Canada; 13 University of Calgary, Calgary, AB, Canada; 14 Primary Care Alberta, Calgary, AB, Canada; 15 Department of Computer Science, Aalborg University, Aalborg, Denmark; 16 Department of Health Science and Technology, Faculty of Medicine, Aalborg University, Aalborg, Denmark; 17 Center for General Practice at Aalborg University, Aalborg, Denmark

In “Evidence for Digital Health Tools Designed to Support the Triage of Musculoskeletal Conditions in Primary, Urgent, and Emergency Care Settings: Scoping Review*”* [1], the authors made two corrections.

In the abstract, the list of databases searched has been amended to remove PsycINFO and Cochrane Library, and these were replaced with Central (OVID) and Compendex. This has also been further specified in the Methods section.

In the abstract, the following sentence was revised:

Systematic searches in MEDLINE (OVID), CINAHL (EBSCO), PsycINFO (EBSCO), Embase (OVID), Cochrane Library, Web of Science, OpenGrey, Google Scholar, arXiv, medRxiv, and an extensive gray literature search were conducted with a librarian scientist from inception to September 18, 2025.

This sentence now reads:

Systematic searches in MEDLINE (OVID), Embase (OVID), CENTRAL (OVID), CINAHL (EBSCO), Compendex, Web of Science, OpenGrey, Google Scholar, arXiv, medRxiv, and an extensive gray literature search were conducted with a librarian scientist from inception to September 18, 2025.

In the Methods section, the following sentence was revised:

An electronic search was conducted in 6 databases (MEDLINE [OVID], CINAHL [EBSCO], PsycINFO [EBSCO], Embase [OVID], the Cochrane Library, and Web of Science) and 4 gray literature sites (OpenGrey, GoogleScholar, arXiv, and medRxiv) with the aid of a biomedical librarian and information specialist.

The sentence now reads:

An electronic search was conducted in 6 databases (MEDLINE [OVID], Embase [OVID], CENTRAL [OVID], CINAHL [EBSCO], Compendex, and Web of Science) and 4 gray literature sites (OpenGrey, GoogleScholar, arXiv, and medRxiv) with the aid of a biomedical librarian and information specialist.

The correction will appear in the online version of the paper on the JMIR Publications website, together with the publication of this correction notice. Because this was made after submission to PubMed, PubMed Central, and other full-text repositories, the corrected article has also been resubmitted to those repositories.
