# Supplementary material for: Evidence for Digital Health Tools Designed to Support the Triage of Musculoskeletal Conditions in Primary, Urgent, and Emergency Care Settings: Scoping Review
Source: J Med Internet Res. 2026 Jan 14;28:e81578. doi: 10.2196/81578 (PMC12803503; doi:10.2196/81578)
Supplement: Multimedia Appendix 1 [file jmir-v28-e81578-s001.docx]

Searches were created iteratively with the research team and conducted by a biomedical librarian (DG).

Complete search strategy of other databases can be found on OSF <https://osf.io/y5rp>

**Database: OVID MEDLINE(R) ALL <1946 to August 13th, 2024>**

| # | Query | Results |
| --- | --- | --- |
| 1 | exp pain/ | 491,716 |
| 2 | (pain* or ache* or aching).ti,ab,kf. | 1,001,401 |
| 3 | (stiff or stiffness or impingement*).ti,ab,kf. | 124,896 |
| 4 | (strain* or sprain*).ti,ab,kf. | 934,300 |
| 5 | or/1-4 | 2,181,517 |
| 6 | exp Musculoskeletal System/ | 1,662,225 |
| 7 | (muscle* or tendon* or ligament*).ti,ab,kf. | 1,045,197 |
| 8 | exp Lower extremity/ | 196,001 |
| 9 | (knee or knees).ti,ab,kf. | 208,905 |
| 10 | (hip or hips).ti,ab,kf. | 195,700 |
| 11 | (ankle or ankles).ti,ab,kf. | 84,099 |
| 12 | (foot or feet).ti,ab,kf. | 154,247 |
| 13 | exp Upper extremity/ | 196,496 |
| 14 | shoulder*.ti,ab,kf. | 102,630 |
| 15 | elbow*.ti,ab,kf. | 45,468 |
| 16 | wrist*.ti,ab,kf. | 53,459 |
| 17 | hand*.ti,ab,kf. | 810,443 |
| 18 | (arm or arms or forearm*).ti,ab,kf. | 308,130 |
| 19 | exp back/ | 23,577 |
| 20 | ((low* or upper) and back).ti,ab,kf. | 100,126 |
| 21 | exp neck/ | 35,412 |
| 22 | neck.ti,ab,kf. | 287,348 |
| 23 | (spine or spinal).ti,ab,kf. | 461,654 |
| 24 | lumbar.ti,ab,kf. | 142,192 |
| 25 | cervical.ti,ab,kf. | 286,656 |
| 26 | Joint*.ti,ab,kf. | 455,005 |
| 27 | exp Musculoskeletal Diseases/ | 1,279,843 |
| 28 | (musculoskeletal injur* or MSK injur*).ti,ab,kf. | 5,323 |
| 29 | (Orthopedic disorder* or orthopaedic disorder*).ti,ab,kf. | 550 |
| 30 | exp "Sprains and Strains"/ | 21,663 |
| 31 | bursitis.ti,ab,kf. | 3,700 |
| 32 | capsulitis.ti,ab,kf. | 1,775 |
| 33 | (tendonitis or tendinitis).ti,ab,kf. | 4,218 |
| 34 | impingement syndrome*.ti,ab,kf. | 3,092 |
| 35 | acute injur*.ti,ab,kf. | 5,480 |
| 36 | anterior cruciate ligament injuries/ | 15,018 |
| 37 | ACL injur*.ti,ab,kf. | 5,879 |
| 38 | anterior cruciate ligament injur*.ti,ab,kf. | 3,203 |
| 39 | tibial meniscus injuries/ | 5,457 |
| 40 | menisc* injur*.ti,ab,kf. | 2,479 |
| 41 | Whiplash Injuries/ | 3,482 |
| 42 | whiplash*.ti,ab,kf. | 3,541 |
| 43 | rotator cuff injuries/ | 8,835 |
| 44 | (rotator adj3 injur*).ti,ab,kf. | 1,311 |
| 45 | myositis.ti,ab,kf. | 13,925 |
| 46 | myopath*.ti,ab,kf. | 35,047 |
| 47 | Gout/ | 13,570 |
| 48 | Gout.ti,ab,kf. | 17,656 |
| 49 | arthritis/ | 38,060 |
| 50 | Arthritis.ti,ab,kf. | 229,938 |
| 51 | Osteoarthr*.ti,ab,kf. | 111,506 |
| 52 | Arthralgia*.ti,ab,kf. | 11,444 |
| 53 | Arthrosis.ti,ab,kf. | 5,989 |
| 54 | Pain syndrome*.ti,ab,kf. | 21,345 |
| 55 | Rheumatic diseases/ | 26,022 |
| 56 | Rheum*.ti,ab,kf. | 235,565 |
| 57 | or/6-26 | 4,556,448 |
| 58 | 5 and 57 | 557,936 |
| 59 | or/27-56,58 | 1,876,509 |
| 60 | exp Telemedicine/ | 53,608 |
| 61 | (Tele*medicine or telemedicine).ti,ab,kf. | 31,006 |
| 62 | Tele*monitor*.ti,ab,kf. | 3,041 |
| 63 | tele*care.ti,ab,kf. | 1,274 |
| 64 | Tele*health.ti,ab,kf. | 19,194 |
| 65 | Tele*consult*.ti,ab,kf. | 2,753 |
| 66 | tele*emergency.ti,ab,kf. | 2 |
| 67 | (tele-referral* or telereferral*).ti,ab,kf. | 15 |
| 68 | (tele-rehab* or telerehab*).ti,ab,kf. | 3,368 |
| 69 | Virtual care.ti,ab,kf. | 1,948 |
| 70 | Virtual medicine.ti,ab,kf. | 104 |
| 71 | Virtual consult*.ti,ab,kf. | 548 |
| 72 | remote consultation/ | 6,116 |
| 73 | remote monitoring.ti,ab,kf. | 5,772 |
| 74 | distant monitoring.ti,ab,kf. | 9 |
| 75 | patient monitoring.ti,ab,kf. | 6,045 |
| 76 | Internet/ | 86,475 |
| 77 | Multimedia/ | 2,321 |
| 78 | Medical Informatics/ | 13,392 |
| 79 | Computers, Handheld/ | 4,246 |
| 80 | mobile health.ti,ab,kf. | 11,907 |
| 81 | electronic health.ti,ab,kf. | 43,311 |
| 82 | (E-health or ehealth).ti,ab,kf. | 13,879 |
| 83 | (M-health or mhealth).ti,ab,kf. | 13,525 |
| 84 | internet.ti,ab,kf. | 87,382 |
| 85 | computer interface*.ti,ab,kf. | 10,065 |
| 86 | user interface*.ti,ab,kf. | 10,322 |
| 87 | multimedia.ti,ab,kf. | 7,309 |
| 88 | Cell*phone.ti,ab,kf. | 585 |
| 89 | mobile telephone*.ti,ab,kf. | 616 |
| 90 | mobile phone*.ti,ab,kf. | 17,382 |
| 91 | mobile app*.ti,ab,kf. | 14,060 |
| 92 | mobile device*.ti,ab,kf. | 5,971 |
| 93 | smart phone*.ti,ab,kf. | 1,916 |
| 94 | telephone screen*.ti,ab,kf. | 332 |
| 95 | tablet app*.ti,ab,kf. | 400 |
| 96 | clinical informatics.ti,ab,kf. | 920 |
| 97 | medical computer science*.ti,ab,kf. | 24 |
| 98 | medical data.ti,ab,kf. | 8,876 |
| 99 | medical informatics.ti,ab,kf. | 4,557 |
| 100 | medical information science*.ti,ab,kf. | 55 |
| 101 | medical information technolog*.ti,ab,kf. | 73 |
| 102 | health informatics.ti,ab,kf. | 4,412 |
| 103 | health information technolog*.ti,ab,kf. | 4,722 |
| 104 | ipad*.ti,ab,kf. | 2,244 |
| 105 | laptop*.ti,ab,kf. | 2,919 |
| 106 | tablet computer*.ti,ab,kf. | 1,134 |
| 107 | hand held computer*.ti,ab,kf. | 244 |
| 108 | (Web-based or web based).ti,ab,kf. | 51,582 |
| 109 | *artificial intelligence/ | 34,939 |
| 110 | (Artificial intelligence or AI).ti,kf. | 67,313 |
| 111 | deep learning.ti,kf. | 60,337 |
| 112 | machine learning.ti,kf. | 97,101 |
| 113 | hierarchical learning.ti,kf. | 50 |
| 114 | natural language processing.ti,kf. | 6,860 |
| 115 | Computational Intelligence.ti,kf. | 332 |
| 116 | Computer reasoning.ti,kf. | 4 |
| 117 | digital health.ti,ab,kf. | 14,594 |
| 118 | Digital health technolog*.ti,ab,kf. | 1,822 |
| 119 | (digital and (assess* or tool*)).ti,ab,kf. | 74,064 |
| 120 | Digital care.ti,ab,kf. | 349 |
| 121 | or/60-120 | 640,919 |
| 122 | triage/ | 16,273 |
| 123 | Triage.ti,ab,kf. | 29,247 |
| 124 | Digital triage.ti,ab,kf. | 45 |
| 125 | (Tele-triage or teletriage).ti,ab,kf. | 78 |
| 126 | Virtual triage.ti,ab,kf. | 33 |
| 127 | Decision making/ | 110,582 |
| 128 | Clinical decision-making/ | 16,573 |
| 129 | Clinical reasoning/ | 874 |
| 130 | Diagnosis/ | 17,577 |
| 131 | Decision support techniques/ | 23,700 |
| 132 | Decision making computer-assisted/ | 0 |
| 133 | Computer-Assisted Decision Making/ | 2,889 |
| 134 | Algorithms/ | 335,759 |
| 135 | Decision making.ti,ab,kf. | 248,020 |
| 136 | Clinical decision-making.ti,ab,kf. | 37,374 |
| 137 | Clinical reason*.ti,ab,kf. | 6,930 |
| 138 | Diagno*.ti,ab,kf. | 3,492,999 |
| 139 | Decision support technique*.ti,ab,kf. | 660 |
| 140 | Decision making computer-assisted.ti,ab,kf. | 45 |
| 141 | Computer-Assisted Decision Making.ti,ab,kf. | 110 |
| 142 | Algorithm*.ti,ab,kf. | 442,512 |
| 143 | Decision Analys*.ti,ab,kf. | 6,963 |
| 144 | Decision Aid*.ti,ab,kf. | 5,378 |
| 145 | Decision Model*.ti,ab,kf. | 3,549 |
| 146 | Decision Support Model.ti,ab,kf. | 217 |
| 147 | self-assess*.ti,ab,kf. | 23,651 |
| 148 | self-refer*.ti,ab,kf. | 6,572 |
| 149 | self-consult*.ti,ab,kf. | 1 |
| 150 | (symptom* adj5 checker*).mp. | 221 |
| 151 | symptom* evaluation.ti,ab,kf. | 1,600 |
| 152 | symptom* assessment.ti,ab,kf. | 5,349 |
| 153 | Symptom assessment/ | 7,288 |
| 154 | or/122-153 | 4,348,908 |
| 155 | 58 and 121 and 154 | 1,868 |

**Database: Updated Search with same search terms <August 13th, 2024 to September 18, 2025>**

Please refer to uploaded search strategy on OSF for complete search terms <https://osf.io/y5rp>

**BEFORE DUPLICATES**

**OVID MEDLINE=347**

**EMBASE=355**

**CENTRAL=44**

**WEB OF SCIENCE=11**

**SCOPUS=0**

**AFTER DUPLICATES**

**N=675 new records identified**
