# Supplementary material for: Evidence for Digital Health Tools Designed to Support the Triage of Musculoskeletal Conditions in Primary, Urgent, and Emergency Care Settings: Scoping Review
Source: J Med Internet Res. 2026 Jan 14;28:e81578. doi: 10.2196/81578 (PMC12803503; doi:10.2196/81578)
Supplement: Multimedia Appendix 2 [file jmir-v28-e81578-s002.docx]

**Appendix 2: Grey literature Search Strategy and Results**

Currently, there is no standardized way to conduct grey literature searches but searches should be systematically conducted and transparently reported. In addition to reviewing OpenGrey, GoogleScholar, arXiv.org, and medRxiv.org, we also reviewed relevant journal, agencies, conferences, and government sites. Our team has an extensive background and knowledge-base that expands several international countries, digital health expertise, health systems expertise, and clinical trial expertise of musculoskeletal/advance practice physios in emergency settings. Based on our screening and search strategy, we identified digital, triage and musculoskeletal/MSK as keywords for grey literature sources that would most likely yield relevant papers to our study. We reviewed public-facing websites of major health organizations in Australia, Canada, and England. We chose to include these health systems as they have a similar universal-coverage model, and the goal of our review is to inform future work in the Canadian health system context.

We defined grey literature as non-peer reviewed work, understanding some non-peered reviewed work may filter into our main search. We used ResearchRabbit© (Seattle, USA) (free AI-powered online platform used for citation-based literature mapping tool) to identify additional sources. Google Scholar was searched using the keywords described above and the first 10 pages returned were reviewed. UK, Can, Australia government sites were searched using the search bar to identify relevant documents. Finally, relevant journal websites and conferences identified by team members were searched using the keywords and only articles published in the last 10 years (2014-2024) were reviewed (Table below). We chose the last 10 years to manage the feasibly of the search returns. Records identified from ResearchRabbit were cross-referenced to identify duplicates. From this, 271 unique records were further identified to be screened.

| **Key Journals (last 10 years)** | **Website** |
| --- | --- |
| Journal of Medical Internet Research | <https://www.jmir.org> |
| Nature npj Digital Medicine | <https://www.nature.com/npjdigitalmed/> |
| JMIR mHealth and uHealth | <https://www.mhealth.jmir.org> |
| Lancet Digital Health | <https://www.thelancet.com/journals/landig/home> |
| BMJ Health & Care Informatics | <https://bmjdigitalhealth.bmj.com/> |
| PLOS Digital Health | https://journals.plos.org/digitalhealth/ |
| IEEE Xplore | <https://ieeexplore.ieee.org/xpl/RecentIssue.jsp?punumber=6221020> |
| Journal of the American Medical Informatics Association | https://academic.oup.com/jamia |
| International Journal of Medical Informatics | https://www.sciencedirect.com/journal/international-journal-of-medical-informatics |
| Canadian Journal of Health Technologies | http://canjhealthtechnol.ca/index.php/cjht |
| JAMA Network Open | <https://jamanetwork.com/journals/jamanetworkopen> |
| ACM Transactions on Computing for Healthcare | <https://dl.acm.org/journal/health.> |
| **Key Conferences** | **Website (preferably link to abstracts/posters)** |
| Digital health world congress | <https://digitalhealthcareworldcongress.com/agenda/> |
| HIMSS Global Health Conference & Exhibition | <https://www.himssconference.com/education-topics/> |
| International Association for the Study of Pain World Congress | <https://www.worldcongress2024.org/event/8a72888b-ef45-44a3-946e-14ca434af118/websitePage:c220c5b4-d46e-4884-9370-58f58ad79cc7?session=df6b8d12-29a7-4c0b-b413-6f4569690917> |
|  |  |
|  |  |
|  |  |
| **Government Facing Websites** | **Website** |
| Digtal Health Canada | <https://digitalhealthcanada.com/> |
| Canada Health Infoway | <https://www.infoway-inforoute.ca/en/> |
| Canadian Institute for Health Information (CIHI) | <https://www.cihi.ca/en> |
| Centre for advancing healht outcomes |  |
| National Health Servies UK | <https://digital.nhs.uk/> |
| Australia Digital Health | digitalhealth.gov.au |
| **Societies** | **Website** |
| International Society for Telemedicine and eHealth | <https://www.isfteh.org/events> |
| Healthcare Information and Management Systems Society | <https://www.himss.org/> |
| American Medical Informatics Association | <https://amia.org/> |
| DIGITAL HEALTH SOCIETY | <https://echalliance.com/digital-health-society/> |
