# Supplementary material for: Evidence for Digital Health Tools Designed to Support the Triage of Musculoskeletal Conditions in Primary, Urgent, and Emergency Care Settings: Scoping Review
Source: J Med Internet Res. 2026 Jan 14;28:e81578. doi: 10.2196/81578 (PMC12803503; doi:10.2196/81578)
Supplement: Multimedia Appendix 3 [file jmir-v28-e81578-s003.docx]

| Study | Title | Notes |
| --- | --- | --- |
| Bisson 2015 | How Accurate Are Patients At Diagnosing The Cause Of Their Knee Pain With The Help Of A Web-based Symptom Checker? | Exclusion reason: 10. duplicate or data is duplicate |
| Knevel 2021 | RHEUMATIC? - A DIGITAL DIAGNOSTIC DECISION SUPPORT TOOL FOR INDIVIDUALS SUSPECTING RHEUMATIC DISEASES: A MULTICENTER VALIDATION STUDY | Exclusion reason: 10. duplicate or data is duplicate |
| Knitza 2020 | REAL-WORLD EFFECTIVENESS AND PERCEIVED USEFULNESS OF SYMPTOM CHECKERS IN RHEUMATOLOGY: INTERIM REPORT FROM THE PROSPECTIVE MULTICENTER BETTER STUDY | Exclusion reason: 10. duplicate or data is duplicate |
| Knitza 2023 | ACCURACY OF AN AI-BASED SYMPTOM CHECKER AND AN ONLINE SELF-REFERRAL TOOL IN RHEUMATOLOGY: RESULTS FROM A MULTICENTER RANDOMIZED CONTROLLED TRIAL | Exclusion reason: 10. duplicate or data is duplicate |
| Qin 2025 | DEVELOPMENT AND VALIDATION OF ‘RHEUMATIC ‘ - A DIGITAL SYMPTOM CHECKER FOR CONFIRMATION AND PREDICTION OF RHEUMATIC DISEASES: A LARGE-SCALE PROSPECTIVE STUDY | Exclusion reason: 10. duplicate or data is duplicate |
| Salisbury 2013 | A pragmatic randomised controlled trial of the effectiveness and cost-effectiveness of ‘PhysioDirect’ telephone assessment and advice services for physiotherapy | Exclusion reason: 10. duplicate or data is duplicate |
|  | Digital Assessment Routing Tool (DART): pilot Study | Exclusion reason: 10. duplicate or data is duplicate |
| Dlott 2022 | Evaluating musculoskeletal urgent care center triage and transfer of emergency conditions for emergency surgical assessment and intervention | Exclusion reason: 2. not human/human data |
| Ettlin 2016 | Design, construction, and technical implementation of a web-based interdisciplinary symptom evaluation (WISE) - a heuristic proposal for orofacial pain and temporomandibular disorders | Exclusion reason: 2. not human/human data |
| Hwang 2009 | Evaluation of the internet for finding persons with undiagnosed rheumatoid arthritis and systemic lupus erythematosus | Exclusion reason: 2. not human/human data |
| Roch 2025 | Diagnosis, treatment, and prevention of ankle sprains: Comparing free chatbot recommendations with clinical guidelines. | Exclusion reason: 2. not human/human data |
| Bousquet 2022 | Supporting Diagnosis With Next-Generation Artificial Intelligence | Exclusion reason: 3. not relevant study design |
| Harring 2024 | When do medical operators choose to use, or not use, video in emergency calls? A case study | Exclusion reason: 3. not relevant study design |
| Kaji 2024 | Present and Future Optimization of Orthopaedic Care Through Machine Learning Algorithms | Exclusion reason: 3. not relevant study design |
| Murphy 2021 | Assuring safety and efficacy of nurse triage for electronic consultation to improve access to specialty care | Exclusion reason: 3. not relevant study design |
| Shayo 2023 | Expanding access to rehabilitation using mobile health to address musculoskeletal pain and disability | Exclusion reason: 3. not relevant study design |
| Silverman 2010 | The utility and limitations of FRAX: A us perspective | Exclusion reason: 3. not relevant study design |
| Singh 2013 | Knowledge-base medical decision support system for knee pain management | Exclusion reason: 3. not relevant study design |
|  | Digital Health Interventions for Musculoskeletal Pain: A Growth Area | Exclusion reason: 3. not relevant study design |
| Devine 2020 | Development and Evaluation of a Nurse Practitioner-Directed Orthopedic Telemedicine Program | Exclusion reason: 3. not relevant study design |
| Simmich 2023 | Content and Quality of Mobile Apps for the Monitoring of Musculoskeletal or Neuropathic Pain in Australia: Systematic Evaluation | Exclusion reason: 3. not relevant study design |
| Vardell 2011 | Isabel, a Clinical Decision Support System | Exclusion reason: 3. not relevant study design |
| Vomer 2023 | Evaluation of the Hand and Wrist via Telephone and Video Visit | Exclusion reason: 3. not relevant study design |
| Weisberg 2020 | The first use of artificial intelligence (AI) in the ER: triage not diagnosis | Exclusion reason: 3. not relevant study design |
| Zegers 2025 | POS1131 Prediction Models for Rheumatic Diseases: From Clinical Simplicity to Data-Driven Complexity with Patient-reported Symptoms for an Online Symptom Checker | Exclusion reason: 3. not relevant study design |
| Burgess 2025 | Optimising Musculoskeletal Patient Flow Through Digital Triage and Supported Self‐Management: A Service Evaluation Set Within Community Musculoskeletal Care | Exclusion reason: 4. Not adult population; **not all participants were over 18 years old and we could not subgroup data between adult and non adult. |
| Chan 2021 | PERFORMANCE OF A NEW SYMPTOM CHECKER IN PATIENT TRIAGE: CANADIAN COHORT STUDY | Exclusion reason: 4. Not adult population; **not all participants were over 18 years old and we could not subgroup data between adult and non adult. |
| Khan 2025 | An Artificial Intelligence Based Approach to Musculoskeletal Acute Knee Injury Triage | Exclusion reason: 4. Not adult population; **not all participants were over 18 years old and we could not subgroup data between adult and non adult. |
| Lowe 2025 | Improving Safety, Efficiency, Cost, and Satisfaction Across a Musculoskeletal Pathway Using the Digital Assessment Routing Tool for Triage: Quality Improvement Stud | Exclusion reason: 4. Not adult population; **not all participants were over 18 years old and we could not subgroup data between adult and non adult. |
| Abramson 2022 | TRANSFORMING A VA REGIONAL INTEGRATED HEALTH CARE SYSTEM TO MEET URGENT NEEDS WITH JUST-IN-TIME VIRTUAL URGENT CARE | Exclusion reason: 5. not msk population |
| Chetty 2012 | Telephone triage assessment for musculoskeletal disorders: Part 2 | Exclusion reason: 5. not msk population |
| Chetty 2012 | Telephone triage assessment for musculoskeletal disorders: Part 1 | Exclusion reason: 5. not msk population |
| deAraujo 2024 | PAINe: An Artificial Intelligence-based Virtual Assistant to Aid in the Differentiation of Pain of Odontogenic versus Temporomandibular Origin. | Exclusion reason: 5. not msk population |
| Emmert 2023 | A diagnostic support system based on pain drawings: binary and k-disease classification of EDS, GBS, FSHD, PROMM, and a control group with Pain2D | Exclusion reason: 5. not msk population |
| Fraser 2022 | Evaluation of Diagnostic and Triage Accuracy and Usability of a Symptom Checker in an Emergency Department: Observational Study | Exclusion reason: 5. not msk population |
| Frost 2011 | Patient-reported outcomes as a source of evidence in off-label prescribing: analysis of data from PatientsLikeMe | Exclusion reason: 5. not msk population |
| Gellert 2022 | A multinational survey of patient utilization of and value conveyed through virtual symptom triage and healthcare referral | Exclusion reason: 5. not msk population |
| Haque 2021 | A nomogram-based diabetic sensorimotor polyneuropathy severity prediction using Michigan neuropathy screening instrumentations | Exclusion reason: 5. not msk population |
| Inokuchi 2022 | Machine learning models predicting undertriage in telephone triage | Exclusion reason: 5. not msk population |
| Kazi 2017 | Using mobile technology to optimize disease surveillance and healthcare delivery at mass gatherings: a case study from India's Kumbh Mela | Exclusion reason: 5. not msk population |
| Liu 2024 | Triage Accuracy and the Safety of User-Initiated Symptom Assessment With an Electronic Symptom Checker in a Real-Life Setting: Instrument Validation Study. | Exclusion reason: 5. not msk population |
| M 2021 | Creating Mobile Self-Triage Applications: Requirements and Usability Perspectives | Exclusion reason: 5. not msk population |
| Michalowski 2005 | Design and development of a mobile system for supporting emergency triage | Exclusion reason: 5. not msk population |
| Morse 2020 | Use characteristics and triage acuity of a digital symptom checker in a large integrated health system: Population-based descriptive study | Exclusion reason: 5. not msk population |
| N 2009 | Computer Generated Self-Care Advice via Web-Based Triage of Complaints in Primary Care | Exclusion reason: 5. not msk population |
| Nasser 2023 | Evaluating the utility of a remote acuity prediction tool in a Canadian academic emergency department | Exclusion reason: 5. not msk population |
| Nijland 2010 | Patient use and compliance with medical advice delivered by a web-based triage system in primary care | Exclusion reason: 5. not msk population |
| Roivainen 2020 | Telephone triage performed by nurses reduces non-urgent ambulance missions: A prospective observational pilot study in Finland | Exclusion reason: 5. not msk population |
| Ronicke 2019 | Can a decision support system accelerate rare disease diagnosis? Evaluating the potential impact of Ada DX in a retrospective study | Exclusion reason: 5. not msk population |
| Sexton 2023 | Clinician-led secondary triage in England's urgent care delivery: a cross-sectional study | Exclusion reason: 5. not msk population |
| Shahid 2018 | What misdiagnosed patients can tell us about types and origins of diagnostic errors | Exclusion reason: 5. not msk population |
| Silva 2022 | Orthopedic Asynchronous Teleconsultation for Primary Care Patients by a Large-Scale Telemedicine Service in Minas Gerais, Brazil | Exclusion reason: 5. not msk population |
| Turner 2013 | Impact of the urgent care telephone service NHS 111 pilot sites: a controlled before and after study | Exclusion reason: 5. not msk population |
| Verzantvoort 2018 | Self-triage for acute primary care via a smartphone application: Practical, safe and efficient? | Exclusion reason: 5. not msk population |
| Begum 2020 | Can a specialist nurse replace a consultant-an innovative model for osteoporosis care | Exclusion reason: 6. not digital health tool |
| Brennan 2020 | Reduction of Orthopaedic and Rheumatology Outpatient Waiting Lists: The National Musculoskeletal Physiotherapy Triage Initiative | Exclusion reason: 6. not digital health tool |
| Browne 2023 | Development of a Referral Tool for a Musculoskeletal Pain Management Service: A Service Improvement Project | Exclusion reason: 6. not digital health tool |
| Cui 2021 | Effects of telemedicine triage on efficiency and cost-effectiveness in spinal care | Exclusion reason: 6. not digital health tool |
| Field 2016 | CLINICAL OUTCOMES IN A LARGE COHORT OF MUSCULOSKELETAL PATIENTS UNDERGOING CHIROPRACTIC CARE IN THE UNITED KINGDOM: A COMPARISON OF SELF- AND NATIONAL HEALTH SERVICE-REFERRED ROUTES | Exclusion reason: 6. not digital health tool |
| Labinsky 2023 | ADDRESSING AXSPA DIAGNOSTIC DELAY BY IMPLEMENTATION OF ASYNCHRONOUS TELEMEDICINE AND MEDICAL STUDENT-SUPPORTED VISITS | Exclusion reason: 6. not digital health tool |
| Liang 2015 | An Internet-based technique for the identification of persons with symptoms of inflammatory polyarthritis of less than 12 weeks | Exclusion reason: 6. not digital health tool |
| Mackenzie 2018 | Discharged but not dissatisfied: outcomes and satisfaction of patients discharged from the Edinburgh Trauma Triage Clinic | Exclusion reason: 6. not digital health tool |
| Ratzlaff 2012 | Clinical validation of an Internet-based questionnaire for ascertaining hip and knee osteoarthritis | Exclusion reason: 6. not digital health tool |
| VanHooff 2013 | Development of a clinical decision tool to support spine surgeons in the triage of chronic lowback pain patients | Exclusion reason: 6. not digital health tool |
|  | Musculoskeletal triage: Do advanced practitioners agree on referral allocation?...Physiotherapy UK Virtual Conference, November 5-6, 2021 | Exclusion reason: 6. not digital health tool |
| Abel 2024 | An Artificial Intelligence-Based Support Tool for Lumbar Spinal Stenosis Diagnosis from Self-Reported History Questionnaire | Exclusion reason: 7. tool not designed specifically to triage or diagnose |
| Antony 2022 | / #623 PATIENT STATES: ARTIFICIAL INTELLIGENCE-DRIVEN METRIC PROVIDING COMPREHENSIVE YET STRAIGHTFORWARD UNDERSTANDING OF CHRONIC PAIN PATIENTS: TRACK 4: CLINICAL USE OF SCS / DRG | Exclusion reason: 7. tool not designed specifically to triage or diagnose |
| Barghouthi 2012 | The use of a camera-enabled mobile phone to triage patients with nasal bone injuries | Exclusion reason: 7. tool not designed specifically to triage or diagnose |
| Boy 2023 | INTEGRATION OF TELEMEDICINE AND MEDICAL STUDENTS INTO A NEW DIAGNOSTIC PATHWAY FOR PATIENTS WITH SUSPECTED AXSPA: A QUALITATIVE EXPLORATION OF THE PATIENTS' EXPERIENCE | Exclusion reason: 7. tool not designed specifically to triage or diagnose |
| Bradlow 2022 | EARLY DIAGNOSIS OF INFLAMMATORY ARTHRITIS (IA) USING MACHINE LEARNING ANALYSIS OF GP REFERRAL LETTERS AND BLOOD TESTS TO IMPROVE PRE-HOSPITAL REFERRAL TRIAGE | Exclusion reason: 7. tool not designed specifically to triage or diagnose |
| Buchan 2022 | How effective is a local musculoskeletal condition specific management website? A retrospective evaluation of practice | Exclusion reason: 7. tool not designed specifically to triage or diagnose |
| Clemens 2020 | The Multidisciplinary Approach to The Study of Chronic Pelvic Pain (MAPP) Research Network*: Design and implementation of the Symptom Patterns Study (SPS) | Exclusion reason: 7. tool not designed specifically to triage or diagnose |
| DeIpina 2010 | On the design of a CADS for shoulder pain pathology | Exclusion reason: 7. tool not designed specifically to triage or diagnose |
| Dowell 2023 | Physiotherapy-Led Musculoskeletal Telephone Triage and Advice Service: A Valid Option for Patients Referred From the Emergency Department | Exclusion reason: 7. tool not designed specifically to triage or diagnose |
| Elkin 2018 | Artificial Intelligence: Bayesian versus Heuristic Method for Diagnostic Decision Support | Exclusion reason: 7. tool not designed specifically to triage or diagnose |
| Elseddik 2023 | Predicting CTS Diagnosis and Prognosis Based on Machine Learning Techniques | Exclusion reason: 7. tool not designed specifically to triage or diagnose |
| Fan 2024 | XGBoost-SHAP-based interpretable diagnostic framework for knee osteoarthritis: a population-based retrospective cohort study. | Exclusion reason: 7. tool not designed specifically to triage or diagnose |
| Fioratti 2022 | Feasibility, Usability, and Implementation Context of an Internet-Based Pain Education and Exercise Program for Chronic Musculoskeletal Pain: Pilot Trial of the ReabilitaDOR Program | Exclusion reason: 7. tool not designed specifically to triage or diagnose |
| Fourmy 2020 | Can we manage osteoporosis without patients ever seeing a consultant? | Exclusion reason: 7. tool not designed specifically to triage or diagnose |
| Fudickar 2024 | Natural Language Processing of Referral Letters for Machine Learning-Based Triaging of Patients With Low Back Pain to the Most Appropriate Intervention: Retrospective Study | Exclusion reason: 7. tool not designed specifically to triage or diagnose |
| Fujita 2019 | A Tablet-Based App for Carpal Tunnel Syndrome Screening: Diagnostic Case-Control Study | Exclusion reason: 7. tool not designed specifically to triage or diagnose |
| Geerdink 2021 | Optimizing orthopedic trauma care delivery during the COVID-19 pandemic. A closed-loop audit of implementing a virtual fracture clinic and fast-track pathway in a Dutch level 2 trauma center | Exclusion reason: 7. tool not designed specifically to triage or diagnose |
| Giraldez 2019 | Preferred referral protocol for recent onset arthritis in adults from primary care to Rheumatology | Exclusion reason: 7. tool not designed specifically to triage or diagnose |
| Gossec 2018 | An e-health interactive self-assessment website (Sanoia&reg;) in rheumatoid arthritis. A 12-month randomized controlled trial in 320 patients | Exclusion reason: 7. tool not designed specifically to triage or diagnose |
| H 2024 | Optimizing Patient Care Pathways: Impact Analysis of an AI-Assisted Smart Referral System for Musculoskeletal Services | Exclusion reason: 7. tool not designed specifically to triage or diagnose |
| Hannes 2020 | RheumaTool, a novel clinical decision support system for the diagnosis of rheumatic diseases, and its first validation in a retrospective chart analysis | Exclusion reason: 7. tool not designed specifically to triage or diagnose |
| Hodges 2020 | An Internet-Based Consumer Resource for People with Low Back Pain (MyBackPain): Development and Evaluation | Exclusion reason: 7. tool not designed specifically to triage or diagnose |
| Hong 2023 | Validity of a Mobile Application to Diagnose Temporomandibular Disorders | Exclusion reason: 7. tool not designed specifically to triage or diagnose |
| Jones 2021 | 1518‚ÄÉThe Use of OrthoPathway, Decision Support Software, To Reduce Variation in Practice, Optimise Patient Care and Reduce Face-To-Face Appointments in Trauma and Orthopaedics | Exclusion reason: 7. tool not designed specifically to triage or diagnose |
| Kawi 2024 | Retention, adherence, and acceptability testing of a digital health intervention in a 3-group randomized controlled trial for chronic musculoskeletal pain | Exclusion reason: 7. tool not designed specifically to triage or diagnose |
| Kennedy 2023 | Predicting a diagnosis of ankylosing spondylitis using primary care health records-A machine learning approach | Exclusion reason: 7. tool not designed specifically to triage or diagnose |
| Khan 2013 | Asynchronous telemedicine diagnosis of musculoskeletal injuries through a prototype interface in virtual reality environment | Exclusion reason: 7. tool not designed specifically to triage or diagnose |
| Khanum 2024 | Predicting mechanical neck pain intensity in computer professionals using machine learning: identification and correlation of key features | Exclusion reason: 7. tool not designed specifically to triage or diagnose |
| Khursheed 2024 | Bridging distances and saving costs: insights from a pilot project of telerheumatology in a rural area of Pakistan | Exclusion reason: 7. tool not designed specifically to triage or diagnose |
| Kim 2017 | Computer learning (artificial intelligence) to create a computer-based triage tool classifying referrals as inflammatory or non-inflammatory requiring only patient reported information | Exclusion reason: 7. tool not designed specifically to triage or diagnose |
| Knitza 2022 | Machine learning-based improvement of an online rheumatology referral and triage system | Exclusion reason: 7. tool not designed specifically to triage or diagnose |
| Kokkotis 2022 | Explainable machine learning for knee osteoarthritis diagnosis based on a novel fuzzy feature selection methodology | Exclusion reason: 7. tool not designed specifically to triage or diagnose |
| Kramer 2024 | A COMPARATIVE ANALYSIS OF MANUAL ALLOCATION VS. AUTOMATED AND AI-SUPPORTED APPROACHES FOR INITIAL APPOINTMENT ALLOCATION IN RHEUMATOLOGY | Exclusion reason: 7. tool not designed specifically to triage or diagnose |
| Krebs 2023 | Developing a triage predictive model for access to a spinal surgeon using clinical variables and natural language processing of radiology reports | Exclusion reason: 7. tool not designed specifically to triage or diagnose |
| Lalloo 2014 | Pain-QuILT: Clinical Feasibility of a Web-Based Visual Pain Assessment Tool in Adults With Chronic Pain | Exclusion reason: 7. tool not designed specifically to triage or diagnose |
| Lamper 2022 | An ecoach-pain for patients with chronic musculoskeletal pain in interdisciplinary primary care: A feasibility study...Pain Science in Motion IV Congress 2022, May 19-20th, Maastricht, Netherlands | Exclusion reason: 7. tool not designed specifically to triage or diagnose |
| Lau 2012 | Utilization and cost of a new model of care for managing acute knee injuries: the Calgary acute knee injury clinic | Exclusion reason: 7. tool not designed specifically to triage or diagnose |
| Lewkowicz 2022 | Digital Therapeutic Care Apps With Decision-Support Interventions for People With Low Back Pain in Germany: Cost-Effectiveness Analysis | Exclusion reason: 7. tool not designed specifically to triage or diagnose |
| Li 2023 | Machine learning model successfully identifies important clinical features for predicting outpatients with rotator cuff tears | Exclusion reason: 7. tool not designed specifically to triage or diagnose |
| Lim 2019 | A Deep Neural Network-Based Method for Early Detection of Osteoarthritis Using Statistical Data | Exclusion reason: 7. tool not designed specifically to triage or diagnose |
| Lindsay 2020 | A service evaluation of e-triage in the osteoporosis outpatient clinic-an effective tool to improve patient access? | Exclusion reason: 7. tool not designed specifically to triage or diagnose |
| Lopes 2021 | User-Centered Development of a Mobile App for Biopsychosocial Pain Assessment in Adults: Usability, Reliability, and Validity Study | Exclusion reason: 7. tool not designed specifically to triage or diagnose |
| LopezdeIpina 2010 | A computer-aided decision support system for shoulder pain pathology | Exclusion reason: 7. tool not designed specifically to triage or diagnose |
| Maarseveen 2024 | EMPLOYING MACHINE LEARNING TO PREDICT RA DIAGNOSIS FROM REFERRAL LETTERS BY THE GENERAL PRACTITIONER | Exclusion reason: 7. tool not designed specifically to triage or diagnose |
| Maarseveen 2025 | Improving musculoskeletal care with AI enhanced triage through data driven screening of referral letters | Exclusion reason: 7. tool not designed specifically to triage or diagnose |
| Maghsoud-Lou 2017 | Protocol-Driven Decision Support within e-Referral Systems to Streamline Patient Consultation, Triaging and Referrals from Primary Care to Specialist Clinics | Exclusion reason: 7. tool not designed specifically to triage or diagnose |
| Mao 2025 | Automated diagnosis and classification of temporomandibular joint degenerative disease via artificial intelligence using CBCT imaging. | Exclusion reason: 7. tool not designed specifically to triage or diagnose |
| Marcaccini 2025 | Breaking Bones, Breaking Barriers: ChatGPT, DeepSeek, and Gemini in Hand Fracture Management | Exclusion reason: 7. tool not designed specifically to triage or diagnose |
| Martin 2019 | A randomized controlled trial of online symptom searching to inform patient generated differential diagnoses | Exclusion reason: 7. tool not designed specifically to triage or diagnose |
| Martin 2024 | 'It will get better soon' A retrospective cohort study of the digital physiotherapeutic management of Emergency Department soft tissue injuries | Exclusion reason: 7. tool not designed specifically to triage or diagnose |
| Masieh 2021 | Can a specialist nurse lead a comprehensive osteoporosis service with remote supervision-an innovative concept | Exclusion reason: 7. tool not designed specifically to triage or diagnose |
| Mathew 1988 | Artificial intelligence in the diagnosis of low-back pain and sciatica | Exclusion reason: 7. tool not designed specifically to triage or diagnose |
| McIlroy 2016 | ARE ONLINE SYMPTOM CHECKERS EFFECTIVE FOR PATIENTS WITH INFLAMMATORY ARTHRITIS: A COHORT STUDY | Exclusion reason: 7. tool not designed specifically to triage or diagnose |
| Meron 2023 | Feasibility and acceptability of a telemedicine triage model among Medicaid patients with low back pain referred to a spine center | Exclusion reason: 7. tool not designed specifically to triage or diagnose |
| Mruthyunjaya 2025 | Right Diagnoses But Wrong Reasoning: Current Large-Language Model-Based Agentic Frameworks Have Flawed Clinical Reasoning Despite High Diagnostic Accuracy | Exclusion reason: 7. tool not designed specifically to triage or diagnose |
| Osborne 2013 | Chronic disease self-management (CDSM) for people with musculoskeletal and mental health problems: Steppingup-a new flexible web-based system to serve patients, clinicians and organisations | Exclusion reason: 7. tool not designed specifically to triage or diagnose |
| Perez-Sancristobal 2025 | ABS0413 LET'S ASK THE PATIENT: DISEASE PREDICTION BASED ON FREE-TEXT SYMPTOM DESCRIPTIONS | Exclusion reason: 7. tool not designed specifically to triage or diagnose |
| Powley 2016 | Are online symptoms checkers useful for patients with inflammatory arthritis? | Exclusion reason: 7. tool not designed specifically to triage or diagnose |
| Pujalte 2021 | Cervical Spine Evaluation by Telephone and Video Visit | Exclusion reason: 7. tool not designed specifically to triage or diagnose |
| Qin 2025 | DEVELOPMENT AND VALIDATION OF ‘RHEUMATIC ‘ - A DIGITAL SYMPTOM CHECKER FOR CONFIRMATION AND PREDICTION OF RHEUMATIC DISEASES: A LARGE-SCALE PROSPECTIVE STUDY | Exclusion reason: 7. tool not designed specifically to triage or diagnose |
| Redeker 2023 | IDENTIFICATION OF A DIAGNOSTIC MODEL FOR AXIAL SPONDYLOARTHRITIS IN DAILY CLINICAL PRACTICE USING A RANDOM FOREST MACHINE LEARNING APPROACH | Exclusion reason: 7. tool not designed specifically to triage or diagnose |
| Redeker 2024 | Identification of a machine learning-based diagnostic model for axial spondyloarthritis in rheumatological routine care using a random forest approach. | Exclusion reason: 7. tool not designed specifically to triage or diagnose |
| Rostom 2018 | Improving Access to Rheumatologists: Use and Benefits of an Electronic Consultation Service | Exclusion reason: 7. tool not designed specifically to triage or diagnose |
| Saif 2023 | IMPROVING THE BIOPSYCHOSOCIAL ASSESSMENT AND MANAGEMENT OF PATIENTS WITH FIBROMYALGIA: DEVELOPING A DIGITAL PRE-CLINIC ASSESSMENT TOOL | Exclusion reason: 7. tool not designed specifically to triage or diagnose |
| Salmeron 2017 | Medical diagnosis of Rheumatoid Arthritis using data driven PSO-FCM with scarce datasets | Exclusion reason: 7. tool not designed specifically to triage or diagnose |
| Scheibe 2013 | Efficiency gains for rheumatology consultation from an electronic referral system in a safety net health system | Exclusion reason: 7. tool not designed specifically to triage or diagnose |
| Selani 2024 | PREDICTING FIBROMYALGIA OUTCOME FROM A RHEUMATIC-SPECIFIC SURVEY | Exclusion reason: 7. tool not designed specifically to triage or diagnose |
| Shapiro 2023 | Evaluation of a machine learning tool for the early identification of patients with undiagnosed psoriatic arthritis - A retrospective population-based study | Exclusion reason: 7. tool not designed specifically to triage or diagnose |
| Siebelt 2021 | Machine learning algorithms trained with pre-hospital acquired history-taking data can accurately differentiate diagnoses in patients with hip complaints | Exclusion reason: 7. tool not designed specifically to triage or diagnose |
| Slattery 2022 | Service evaluation of telehealth in a physiotherapy musculoskeletal setting: Patient outcomes and results from risk stratification | Exclusion reason: 7. tool not designed specifically to triage or diagnose |
| Spasic 2020 | Patient Triage by Topic Modeling of Referral Letters: Feasibility Study | Exclusion reason: 7. tool not designed specifically to triage or diagnose |
| Stanley 2023 | An automated, web-based triage tool may optimise referral pathways in elective orthopaedic surgery: A proof-of-concept study | Exclusion reason: 7. tool not designed specifically to triage or diagnose |
| Torad 2024 | Identifying Predictors of Neck Disability in Patients with Cervical Pain Using Machine Learning Algorithms: A Cross-Sectional Correlational Study | Exclusion reason: 7. tool not designed specifically to triage or diagnose |
| Ullah 2023 | Machine learning-based prediction of osteoporosis in postmenopausal women with clinical examined features: A quantitative clinical study | Exclusion reason: 7. tool not designed specifically to triage or diagnose |
| Umapathy 2015 | My joint pain: Web-based osteoarthritis management resource improves quality of care | Exclusion reason: 7. tool not designed specifically to triage or diagnose |
| Umapathy 2015 | The Web-Based Osteoarthritis Management Resource My Joint Pain Improves Quality of Care: A Quasi-Experimental Study | Exclusion reason: 7. tool not designed specifically to triage or diagnose |
| Valsecchi 2024 | RWD127 Preliminary Results of a Machine Learning Model for the Detection of Undiagnosed Cases of Ankylosing Spondylitis in Italy | Exclusion reason: 7. tool not designed specifically to triage or diagnose |
| vanHooff 2014 | The Nijmegen decision tool for chronic low back pain. Development of a clinical decision tool for secondary or tertiary spine care specialists | Exclusion reason: 7. tool not designed specifically to triage or diagnose |
| Venerito 2024 | Large language model-driven sentiment analysis for facilitating fibromyalgia diagnosis | Exclusion reason: 7. tool not designed specifically to triage or diagnose |
| Wallnofer 2024 | Developing and testing a framework for coding general practitioners' free-text diagnoses in electronic medical records - a reliability study for generating training data in natural language processing | Exclusion reason: 7. tool not designed specifically to triage or diagnose |
| Wathall 2019 | Health informatics (HI) innovations in randomised trials and clinical cohorts-identification, screening, stratified care and data collection during primary care consultations | Exclusion reason: 7. tool not designed specifically to triage or diagnose |
| Westley 2022 | Accuracy of virtual assessment in hand trauma | Exclusion reason: 7. tool not designed specifically to triage or diagnose |
| Wu 2025 | Diagnostic Accuracy and Interobserver Reliability of Rotator Cuff Tear Detection With Ultrasonography Are Improved With Attentional Deep Learning. | Exclusion reason: 7. tool not designed specifically to triage or diagnose |
| Yildiz 2024 | An investigation of machine learning algorithms for prediction of temporomandibular disorders by using clinical parameters. | Exclusion reason: 7. tool not designed specifically to triage or diagnose |
| Yu 2016 | Weighted cumulative exposure models helped identify an association between early knee-pain consultations and future knee OA diagnosis | Exclusion reason: 7. tool not designed specifically to triage or diagnose |
| Zatt 2024 | Using machine learning to classify temporomandibular disorders: a proof of concept. | Exclusion reason: 7. tool not designed specifically to triage or diagnose |
| Zhou 2024 | Evaluating ChatGPT responses in the context of a 53-year-old male with a femoral neck fracture: a qualitative analysis | Exclusion reason: 7. tool not designed specifically to triage or diagnose |
| Gymer 2024 | A Case Report: Digital Musculoskeletal Triage and Rehabilitation Tools Improve Outcomes and Offer a Positive Experience for Lower Back Pain | Exclusion reason: 7. tool not designed specifically to triage or diagnose |
| Ryan 2024 | Analgesia use in people with low back pain self-referring into MSK care via Digital Triage in England. An observational Study | Exclusion reason: 7. tool not designed specifically to triage or diagnose |
| Santosa 2023 | Chatbots in SINGAPORE-SCREENING and triaging: Use of a chatbot for screening of rheumatic conditions in a multi-ethnic Asian population | Exclusion reason: 7. tool not designed specifically to triage or diagnose |
| Barroso 2019 | Mobile Application Development for Monitoring Functional Overload and Osteomuscular Lesions | Exclusion reason: 9. Unable to retrieve full text |
|  | Evaluation of Triage Tools in Rheumatology | Exclusion reason: 9. Unable to retrieve full text |

REFERENCES

1. Evaluation of Triage Tools in Rheumatology. 2019

2. Digital Assessment Routing Tool (DART): pilot Study. 2021

3. Digital Health Interventions for Musculoskeletal Pain: A Growth Area. 2022;37(11) doi: 10.1097/01.BACK.0000896768.75483.83

4. Abel F, Garcia E, Andreeva V, et al. An Artificial Intelligence-Based Support Tool for Lumbar Spinal Stenosis Diagnosis from Self-Reported History Questionnaire. 2024;181(101528275) doi: https://dx.doi.org/10.1016/j.wneu.2023.11.020

5. Abramson A, Smith JE. TRANSFORMING A VA REGIONAL INTEGRATED HEALTH CARE SYSTEM TO MEET URGENT NEEDS WITH JUST-IN-TIME VIRTUAL URGENT CARE. 2022;37(Supplement 2) doi: https://dx.doi.org/10.1007/s11606-022-07653-8

6. Antony A, Rauck R, Loudermilk E, et al. / #623 PATIENT STATES: ARTIFICIAL INTELLIGENCE-DRIVEN METRIC PROVIDING COMPREHENSIVE YET STRAIGHTFORWARD UNDERSTANDING OF CHRONIC PAIN PATIENTS: TRACK 4: CLINICAL USE OF SCS / DRG. 2022;25(7 Supplement) doi: https://dx.doi.org/10.1016/j.neurom.2022.08.086

7. Aziz KS. An Artificial Intelligence Based Approach to Musculoskeletal Acute Knee Injury Triage. *Journal of ISAKOS* 2025;12(Supplement 1):100743. doi: https://dx.doi.org/10.1016/j.jisako.2025.100743

8. Barghouthi T, Glynn F, Speaker RB, et al. The use of a camera-enabled mobile phone to triage patients with nasal bone injuries. 2012;18(2) doi: http://dx.doi.org/10.1089/tmj.2011.0112

9. Barroso MP, Ferraz ASM, Oliveira TVR, et al. Mobile Application Development for Monitoring Functional Overload and Osteomuscular Lesions. 2019

10. Begum J, Fourmy J, Nisar MK. Can a specialist nurse replace a consultant-an innovative model for osteoporosis care. 2020;79(SUPPL 1) doi: https://dx.doi.org/10.1136/annrheumdis-2020-eular.1986

11. Bisson LJ, Komm J, Bernas GA, et al. How accurate are patients at diagnosing the cause of their knee pain with the help of a web-based symptom checker? 2015;3(7 Supplement 2) doi: https://dx.doi.org/10.1177/2325967115S00150

12. Bousquet C, Coulet A. Supporting Diagnosis With Next-Generation Artificial Intelligence. 2022;327(14) doi: https://dx.doi.org/10.1001/jama.2022.2303

13. Boy K, May S, Labinsky H, et al. INTEGRATION OF TELEMEDICINE AND MEDICAL STUDENTS INTO A NEW DIAGNOSTIC PATHWAY FOR PATIENTS WITH SUSPECTED AXSPA: A QUALITATIVE EXPLORATION OF THE PATIENTS' EXPERIENCE. 2023;82(Supplement 1) doi: https://dx.doi.org/10.1136/annrheumdis-2023-eular.1678

14. Bradlow A, Wang B, Li W, et al. EARLY DIAGNOSIS OF INFLAMMATORY ARTHRITIS (IA) USING MACHINE LEARNING ANALYSIS OF GP REFERRAL LETTERS AND BLOOD TESTS TO IMPROVE PRE-HOSPITAL REFERRAL TRIAGE. 2022;61(SUPPL 1) doi: https://dx.doi.org/10.1093/rheumatology/keac133.108

15. Brennan A, Ashton J, Callanan E, et al. Reduction of Orthopaedic and Rheumatology Outpatient Waiting Lists: The National Musculoskeletal Physiotherapy Triage Initiative. 2020;113(8)

16. Browne S, Ryan C. Development of a Referral Tool for a Musculoskeletal Pain Management Service: A Service Improvement Project. 2023(53)

17. Buchan S. How effective is a local musculoskeletal condition specific management website? A retrospective evaluation of practice. 2022;114(Supplement 1) doi: https://dx.doi.org/10.1016/j.physio.2021.12.035

18. Burgess R, Tucker K, Smithson R, et al. Optimising Musculoskeletal Patient Flow Through Digital Triage and Supported Self-Management: A Service Evaluation Set Within Community Musculoskeletal Care. *Musculoskeletal Care* 2024;22(4):e70013. doi: https://doi.org/10.1002/msc.70013

19. Chan F, Lai S, Pieterman M, et al. Performance of a new symptom checker in patient triage: Canadian cohort study. *PLOS ONE* 2021;16(12):e0260696. doi: 10.1371/journal.pone.0260696

20. Chetty L. Telephone triage assessment for musculoskeletal disorders: Part 1. 2012;21(20) doi: http://dx.doi.org/10.12968/bjon.2012.21.20.1224

21. Chetty L. Telephone triage assessment for musculoskeletal disorders: Part 2. 2012;21(22) doi: http://dx.doi.org/10.12968/bjon.2012.21.22.1316

22. Clemens JQ, Kutch JJ, Mayer EA, et al. The Multidisciplinary Approach to The Study of Chronic Pelvic Pain (MAPP) Research Network*: Design and implementation of the Symptom Patterns Study (SPS). 2020;39(6) doi: https://dx.doi.org/10.1002/nau.24423

23. Cui S, Sedney CL, Daffner SD, et al. Effects of telemedicine triage on efficiency and cost-effectiveness in spinal care. 2021;21(5) doi: https://dx.doi.org/10.1016/j.spinee.2021.01.006

24. de Araujo BMdM, de Jesus Freitas PF, Deliga Schroder AG, et al. PAINe: An Artificial Intelligence-based Virtual Assistant to Aid in the Differentiation of Pain of Odontogenic versus Temporomandibular Origin. *Journal of endodontics* 2024;50(12):1761-65.e2. doi: https://dx.doi.org/10.1016/j.joen.2024.09.008

25. De Ipina KL, Hernandez MC, Martinez E, et al. On the design of a CADS for shoulder pain pathology. 2010;6076 LNAI(PART 1) doi: 10.1007/978-3-642-13769-3_62

26. Devine CS. Development and Evaluation of a Nurse Practitioner-Directed Orthopedic Telemedicine Program. 2020

27. Dlott CC, Metcalfe T, Khunte A, et al. Evaluating musculoskeletal urgent care center triage and transfer of emergency conditions for emergency surgical assessment and intervention. 2022;101(51) doi: https://dx.doi.org/10.1097/MD.0000000000032519

28. Dowell RJ, Dattani J, Nagy M, et al. Physiotherapy-Led Musculoskeletal Telephone Triage and Advice Service: A Valid Option for Patients Referred From the Emergency Department. 2023;15(2) doi: https://dx.doi.org/10.7759/cureus.34720

29. Elkin P, Schlegel D, Anderson M, et al. Artificial Intelligence: Bayesian versus Heuristic Method for Diagnostic Decision Support. *Applied Clinical Informatics* 2018;09(02):432-39. doi: 10.1055/s-0038-1656547

30. Elseddik M, Mostafa RR, Elashry A, et al. Predicting CTS Diagnosis and Prognosis Based on Machine Learning Techniques. 2023;13(3) doi: https://dx.doi.org/10.3390/diagnostics13030492

31. Emmert D, Szczypien N, Bender TTA, et al. A diagnostic support system based on pain drawings: binary and k-disease classification of EDS, GBS, FSHD, PROMM, and a control group with Pain2D. 2023;18(1) doi: https://dx.doi.org/10.1186/s13023-023-02663-z

32. Ettlin DA, Sommer I, Bronnimann B, et al. Design, construction, and technical implementation of a web-based interdisciplinary symptom evaluation (WISE) - a heuristic proposal for orofacial pain and temporomandibular disorders. 2016;17(1) doi: https://dx.doi.org/10.1186/s10194-016-0670-5

33. Fan Z, Song W, Ke Y, et al. XGBoost-SHAP-based interpretable diagnostic framework for knee osteoarthritis: a population-based retrospective cohort study. *Arthritis research & therapy* 2024;26(1):213. doi: https://dx.doi.org/10.1186/s13075-024-03450-2

34. Field JR, Newell D. CLINICAL OUTCOMES IN A LARGE COHORT OF MUSCULOSKELETAL PATIENTS UNDERGOING CHIROPRACTIC CARE IN THE UNITED KINGDOM: A COMPARISON OF SELF- AND NATIONAL HEALTH SERVICE-REFERRED ROUTES. 2016;39(1) doi: 10.1016/j.jmpt.2015.12.003

35. Fioratti I, Miyamoto GC, Fandim JV, et al. Feasibility, Usability, and Implementation Context of an Internet-Based Pain Education and Exercise Program for Chronic Musculoskeletal Pain: Pilot Trial of the ReabilitaDOR Program. 2022;6(8) doi: 10.2196/35743

36. Fourmy J, Begum J, Nisar MK. Can we manage osteoporosis without patients ever seeing a consultant? 2020;59(Supplement 2) doi: https://dx.doi.org/10.1093/rheumatology/keaa111.087

37. Fraser HSF, Cohan G, Koehler C, et al. Evaluation of Diagnostic and Triage Accuracy and Usability of a Symptom Checker in an Emergency Department: Observational Study. *JMIR Mhealth Uhealth* 2022;10(9):e38364. doi: 10.2196/38364

38. Frost J, Okun S, Vaughan T, et al. Patient-reported outcomes as a source of evidence in off-label prescribing: analysis of data from PatientsLikeMe. 2011;13(1)

39. Fudickar S, Bantel C, Spieker J, et al. Natural Language Processing of Referral Letters for Machine Learning-Based Triaging of Patients With Low Back Pain to the Most Appropriate Intervention: Retrospective Study. 2024;26(100959882) doi: https://dx.doi.org/10.2196/46857

40. Fujita K, Watanabe T, Kuroiwa T, et al. A Tablet-Based App for Carpal Tunnel Syndrome Screening: Diagnostic Case-Control Study. 2019;7(9) doi: https://dx.doi.org/10.2196/14172

41. Geerdink TH, Salentijn DA, De Vries KA, et al. Optimizing orthopedic trauma care delivery during the COVID-19 pandemic. A closed-loop audit of implementing a virtual fracture clinic and fast-track pathway in a Dutch level 2 trauma center. 2021;6(1) doi: https://dx.doi.org/10.1136/tsaco-2021-000691

42. Gellert GA, Orzechowski PM, Price T, et al. A multinational survey of patient utilization of and value conveyed through virtual symptom triage and healthcare referral. 2022;10(101616579) doi: https://dx.doi.org/10.3389/fpubh.2022.1047291

43. Giraldez CR, Espinosa M, Argumanez CM, et al. Preferred referral protocol for recent onset arthritis in adults from primary care to Rheumatology. 2019;78(Supplement 2) doi: https://dx.doi.org/10.1136/annrheumdis-2019-eular.6144

44. Gossec L, Cantagrel A, Soubrier M, et al. An e-health interactive self-assessment website (Sanoia&reg;) in rheumatoid arthritis. A 12-month randomized controlled trial in 320 patients. 2018;85(6) doi: https://doi.org/10.1016/j.jbspin.2017.11.015

45. Gymer M, Guard M, Grinbergs P. A Case Report: Digital Musculoskeletal Triage and Rehabilitation Tools Improve Outcomes and Offer a Positive Experience for Lower Back Pain. 2024;123(Supplement 1) doi: https://dx.doi.org/10.1016/j.physio.2024.04.136

46. Hannes A, Christian M, Johann S, et al. RheumaTool, a novel clinical decision support system for the diagnosis of rheumatic diseases, and its first validation in a retrospective chart analysis. 2020;150(47) doi: https://dx.doi.org/10.4414/smw.2020.20369

47. Haque F, Ibne Reaz MB, Chowdhury MEH, et al. A nomogram-based diabetic sensorimotor polyneuropathy severity prediction using Michigan neuropathy screening instrumentations. 2021;139((Haque, Ibne Reaz, Md Ali, Ashrif A Bakar) Department of Electrical, Electronic and System Engineering, Universiti Kebangsaan Malaysia, Bangi, Selangor 43600, Malaysia(Chowdhury, Rahman) Department of Electrical Engineering, Qatar University, Doha 2713, Q) doi: https://dx.doi.org/10.1016/j.compbiomed.2021.104954

48. Harring AKV, Idland S, Dugstad J. When do medical operators choose to use, or not use, video in emergency calls? A case study. 2024;13(2) doi: https://dx.doi.org/10.1136/bmjoq-2024-002751

49. Hodges PW, Setchell J, Nielsen M. An Internet-Based Consumer Resource for People with Low Back Pain (MyBackPain): Development and Evaluation. 2020;7(1) doi: https://dx.doi.org/10.2196/16101

50. Hong Y-R, Hwangbo N-K, Kim A-H, et al. Validity of a Mobile Application to Diagnose Temporomandibular Disorders. 2023;12(22) doi: https://dx.doi.org/10.3390/jcm12227193

51. Hwang AS, Gall V, Liang MH. Evaluation of the internet for finding persons with undiagnosed rheumatoid arthritis and systemic lupus erythematosus. 2009;15(5) doi: https://dx.doi.org/10.1097/RHU.0b013e3181b126d3

52. Inokuchi R, Iwagami M, Sun Y, et al. Machine learning models predicting undertriage in telephone triage. 2022;54(1) doi: https://dx.doi.org/10.1080/07853890.2022.2136402

53. Jones M, Griffiths-Jones W. 1518 The Use of OrthoPathway, Decision Support Software, To Reduce Variation in Practice, Optimise Patient Care and Reduce Face-To-Face Appointments in Trauma and Orthopaedics. *British Journal of Surgery* 2021;108(Supplement_6):znab259.1060. doi: 10.1093/bjs/znab259.1060

54. Kaji ES, Grove AF, Taunton MJ. Present and Future Optimization of Orthopaedic Care Through Machine Learning Algorithms. 2024;39(5) doi: https://dx.doi.org/10.1016/j.arth.2024.03.043

55. Kawi J, Yeh CH, Grant L, et al. Retention, adherence, and acceptability testing of a digital health intervention in a 3-group randomized controlled trial for chronic musculoskeletal pain. 2024;81 doi: 10.1016/j.ctim.2024.103030

56. Kazi DS, Greenough G, Madhok R, et al. Using mobile technology to optimize disease surveillance and healthcare delivery at mass gatherings: a case study from India's Kumbh Mela. 2017;39(3) doi: 10.1093/pubmed/fdw091

57. Kennedy J, Kennedy N, Cooksey R, et al. Predicting a diagnosis of ankylosing spondylitis using primary care health records-A machine learning approach. 2023;18(3) doi: https://dx.doi.org/10.1371/journal.pone.0279076

58. Khan S, Charissis V, Harrison D, et al. Asynchronous telemedicine diagnosis of musculoskeletal injuries through a prototype interface in virtual reality environment. 2013;8022 LNCS(PART 2) doi: 10.1007/978-3-642-39420-1-6

59. Khanum F, Khan AR, Khan A, et al. Predicting mechanical neck pain intensity in computer professionals using machine learning: identification and correlation of key features. 2024;12(101616579) doi: https://dx.doi.org/10.3389/fpubh.2024.1307592

60. Khursheed T, Rasheed U, Raza UA, et al. Bridging distances and saving costs: insights from a pilot project of telerheumatology in a rural area of Pakistan. 2024;43(8) doi: https://dx.doi.org/10.1007/s10067-024-07037-9

61. Kim C, Bohn T, Ling CX, et al. Computer learning (artificial intelligence) to create a computer-based triage tool classifying referrals as inflammatory or non-inflammatory requiring only patient reported information. 2017;69(Supplement 10)

62. Knevel R, Knitza J, Hensvold A, et al. Rheumatic -A digital diagnostic decision support tool for individuals suspecting rheumatic diseases: A multicenter validation study. 2021;80(SUPPL 1) doi: https://dx.doi.org/10.1136/annrheumdis-2021-eular.1118

63. Knitza J, Janousek L, Kluge F, et al. Machine learning-based improvement of an online rheumatology referral and triage system. *Frontiers in Medicine* 2022;9 doi: 10.3389/fmed.2022.954056

64. Knitza J, Mohn J, Bergmann C, et al. Real-world effectiveness and perceived usefulness of symptom checkers in rheumatology: Interim report from the prospective multicenter better study. 2020;79(SUPPL 1) doi: https://dx.doi.org/10.1136/annrheumdis-2020-eular.1604

65. Knitza J, Tascilar K, Fuchs F, et al. ACCURACY OF AN AI-BASED SYMPTOM CHECKER AND AN ONLINE SELF-REFERRAL TOOL IN RHEUMATOLOGY: RESULTS FROM A MULTICENTER RANDOMIZED CONTROLLED TRIAL. 2023;82(Supplement 1) doi: https://dx.doi.org/10.1136/annrheumdis-2023-eular.1206

66. Kokkotis C, Ntakolia C, Moustakidis S, et al. Explainable machine learning for knee osteoarthritis diagnosis based on a novel fuzzy feature selection methodology. 2022;45(1) doi: https://dx.doi.org/10.1007/s13246-022-01106-6

67. Kramer S, Floege A, Handt S, et al. A COMPARATIVE ANALYSIS OF MANUAL ALLOCATION VS. AUTOMATED AND AI-SUPPORTED APPROACHES FOR INITIAL APPOINTMENT ALLOCATION IN RHEUMATOLOGY. 2024;83(Supplement 1) doi: https://dx.doi.org/10.1136/annrheumdis-2024-eular.646

68. Krebs B, Nataraj A, McCabe E, et al. Developing a triage predictive model for access to a spinal surgeon using clinical variables and natural language processing of radiology reports. *European Spine Journal* 2023 doi: 10.1007/s00586-023-07552-4

69. Labinsky H, Von Rohr S, Horstmann B, et al. ADDRESSING AXSPA DIAGNOSTIC DELAY BY IMPLEMENTATION OF ASYNCHRONOUS TELEMEDICINE AND MEDICAL STUDENT-SUPPORTED VISITS. 2023;82(Supplement 1) doi: https://dx.doi.org/10.1136/annrheumdis-2023-eular.2059

70. Lalloo C, Kumbhare D, Stinson JN, et al. Pain-QuILT: Clinical Feasibility of a Web-Based Visual Pain Assessment Tool in Adults With Chronic Pain. 2014;16(5) doi: 10.2196/jmir.3292

71. Lamper C, Huijnen I, de Mooij M, et al. An ecoach-pain for patients with chronic musculoskeletal pain in interdisciplinary primary care: A feasibility study...Pain Science in Motion IV Congress 2022, May 19-20th, Maastricht, Netherlands. 2022;22 doi: 10.1111/papr.13128

72. Lau BHF, Lafave MR, Mohtadi NG, et al. Utilization and cost of a new model of care for managing acute knee injuries: the Calgary acute knee injury clinic. *BMC Health Services Research* 2012;12(1):445. doi: 10.1186/1472-6963-12-445

73. Lewkowicz D, Wohlbrandt AM, Bottinger E. Digital Therapeutic Care Apps With Decision-Support Interventions for People With Low Back Pain in Germany: Cost-Effectiveness Analysis. 2022;10(2) doi: https://dx.doi.org/10.2196/35042

74. Li C, Alike Y, Hou J, et al. Machine learning model successfully identifies important clinical features for predicting outpatients with rotator cuff tears. 2023;31(7) doi: https://dx.doi.org/10.1007/s00167-022-07298-4

75. Liang MH, Couto MCM, Duarte CCM, et al. An Internet-based technique for the identification of persons with symptoms of inflammatory polyarthritis of less than 12 weeks. 2015;34(3) doi: https://dx.doi.org/10.1007/s10067-014-2796-7

76. Lim J, Kim J, Cheon S. A Deep Neural Network-Based Method for Early Detection of Osteoarthritis Using Statistical Data. 2019; 16(7).

77. Lindsay JR, Lawrenson G, English S. A service evaluation of e-triage in the osteoporosis outpatient clinic-an effective tool to improve patient access? 2020;15(1) doi: https://dx.doi.org/10.1007/s11657-020-0703-1

78. Liu V, Kaila M, Koskela T. Triage Accuracy and the Safety of User-Initiated Symptom Assessment With an Electronic Symptom Checker in a Real-Life Setting: Instrument Validation Study. *JMIR human factors* 2024;11(101666561):e55099. doi: https://dx.doi.org/10.2196/55099

79. Lopes F, Rodrigues M, Silva AG. User-Centered Development of a Mobile App for Biopsychosocial Pain Assessment in Adults: Usability, Reliability, and Validity Study. 2021;9(5) doi: 10.2196/25316

80. Lopez de Ipina K, Hernandez MC, Grana M, et al. A computer-aided decision support system for shoulder pain pathology. 2010;71 doi: 10.1007/978-3-642-12433-4_83

81. Lowe C, Atherton L, Lloyd P, et al. Improving Safety, Efficiency, Cost, and Satisfaction Across a Musculoskeletal Pathway Using the Digital Assessment Routing Tool for Triage: Quality Improvement Study. *Journal of Medical Internet Research* 2025;27:e67269. doi: 10.2196/67269

82. Maarseveen TD, Glas HK, Veris-Van Dieren J, et al. EMPLOYING MACHINE LEARNING TO PREDICT RA DIAGNOSIS FROM REFERRAL LETTERS BY THE GENERAL PRACTITIONER. 2024;83(Supplement 1) doi: https://dx.doi.org/10.1136/annrheumdis-2024-eular.1728

83. Maarseveen TD, Glas HK, Veris-van Dieren J, et al. Improving musculoskeletal care with AI enhanced triage through data driven screening of referral letters. *npj Digital Medicine* 2025;8(1):98. doi: https://dx.doi.org/10.1038/s41746-025-01495-4

84. Mackenzie SP, Carter TH, Jefferies JG, et al. Discharged but not dissatisfied: outcomes and satisfaction of patients discharged from the Edinburgh Trauma Triage Clinic. 2018;100-B(7) doi: https://dx.doi.org/10.1302/0301-620X.100B7.BJJ-2017-1388.R2

85. Maghsoud-Lou E, Christie S, Abidi SR, et al. Protocol-Driven Decision Support within e-Referral Systems to Streamline Patient Consultation, Triaging and Referrals from Primary Care to Specialist Clinics. 2017;41(9) doi: https://dx.doi.org/10.1007/s10916-017-0791-7

86. Mao W-Y, Fang Y-Y, Wang Z-Z, et al. Automated diagnosis and classification of temporomandibular joint degenerative disease via artificial intelligence using CBCT imaging. *Journal of dentistry* 2025;154(hx1, 0354422):105592. doi: https://dx.doi.org/10.1016/j.jdent.2025.105592

87. Marcaccini G, Seth I, Xie Y, et al. Breaking Bones, Breaking Barriers: ChatGPT, DeepSeek, and Gemini in Hand Fracture Management. *Journal of Clinical Medicine* 2025;14(6):1983. doi: https://dx.doi.org/10.3390/jcm14061983

88. Martin L, Liptrot M, Mobberley H, et al. 'It will get better soon' A retrospective cohort study of the digital physiotherapeutic management of Emergency Department soft tissue injuries. 2024;123(Supplement 1) doi: https://dx.doi.org/10.1016/j.physio.2024.04.164

89. Martin S, Quaye E, Schultz S, et al. A randomized controlled trial of online symptom searching to inform patient generated differential diagnoses. *npj Digital Medicine* 2019;2 doi: 10.1038/s41746-019-0183-0

90. Masieh D, Begum J, Fourmy J, et al. Can a specialist nurse lead a comprehensive osteoporosis service with remote supervision-an innovative concept. 2021;80(SUPPL 1) doi: https://dx.doi.org/10.1136/annrheumdis-2021-eular.1069

91. Mathew B, Norris D, Hendry D, et al. Artificial intelligence in the diagnosis of low-back pain and sciatica. 1988;13(2) doi: https://dx.doi.org/10.1097/00007632-198802000-00007

92. McIlroy G, Powley L, Simons G, et al. ARE ONLINE SYMPTOM CHECKERS EFFECTIVE FOR PATIENTS WITH INFLAMMATORY ARTHRITIS: A COHORT STUDY. *Rheumatology* 2016;55(Supplement 1):i128. doi: https://dx.doi.org/10.1093/rheumatology/kew133.017

93. Meron A, Barber K, Stokes D, et al. Feasibility and acceptability of a telemedicine triage model among Medicaid patients with low back pain referred to a spine center. 2023;14(9918335076906676) doi: https://dx.doi.org/10.1016/j.xnsj.2023.100200

94. Michalowski W, Slowinski R, Wilk S, et al. Design and development of a mobile system for supporting emergency triage. 2005;44(1)

95. Morse KE, Ostberg NP, Jones VG, et al. Use characteristics and triage acuity of a digital symptom checker in a large integrated health system: Population-based descriptive study. 2020;22(11) doi: https://dx.doi.org/10.2196/20549

96. Mruthyunjaya P, Verma S, Agarwal A, et al. Right Diagnoses But Wrong Reasoning: Current Large-Language Model-Based Agentic Frameworks Have Flawed Clinical Reasoning Despite High Diagnostic Accuracy. *SSRN* 2025((Mruthyunjaya, Verma, Agarwal, Ahmed) Department of Clinical Immunology and Rheumatology, Kalinga Institute of Medical Sciences, KIIT Deemed-to-be University, Bhubaneswar, India(Mruthyunjaya) RheumaCARE, Bengaluru, India(Maharana, Mandal) Department of Co)

97. Murphy EJ, Tuot DS. Assuring safety and efficacy of nurse triage for electronic consultation to improve access to specialty care. 2021;30(7) doi: https://dx.doi.org/10.1136/bmjqs-2020-012619

98. Nasser L, McLeod S, Hall J. Evaluating the utility of a remote acuity prediction tool in a Canadian academic emergency department. 2023;25(Supplement 1) doi: https://dx.doi.org/10.1007/s43678-023-00508-z

99. Nijland N, Cranen K, Boer H, et al. Patient use and compliance with medical advice delivered by a web-based triage system in primary care. 2010;16(1) doi: https://dx.doi.org/10.1258/jtt.2009.001004

100. Nijland N, Cranen K, Verlinden SFF, et al. Computer Generated Self-Care Advice via Web-Based Triage of Complaints in Primary Care. *eHealth, Telemedicine, and Social Medicine, International Conference on* 2009:129-35. doi: 10.1109/eTELEMED.2009.17

101. Osborne RH, Dodson S, Batterham R, et al. Chronic disease self-management (CDSM) for people with musculoskeletal and mental health problems: Steppingup-a new flexible web-based system to serve patients, clinicians and organisations. 2013;71(SUPPL. 3) doi: https://dx.doi.org/10.1136/annrheumdis-2012-eular.1882

102. Perez - Sancristobal I, Steinz N, Qin L, et al. ABS0413 LET'S ASK THE PATIENT: DISEASE PREDICTION BASED ON FREE-TEXT SYMPTOM DESCRIPTIONS. *Annals of the Rheumatic Diseases* 2025;84(Supplement 1):1513EP-14. doi: https://dx.doi.org/10.1016/j.ard.2025.06.874

103. Powley L, McIlroy G, Simons G, et al. Are online symptoms checkers useful for patients with inflammatory arthritis? *BMC Musculoskeletal Disorders* 2016;17(1):362. doi: 10.1186/s12891-016-1189-2

104. Pujalte G, Loeffert JR, Bertasi TGO, et al. Cervical Spine Evaluation by Telephone and Video Visit. 2021;13(11) doi: https://dx.doi.org/10.7759/cureus.19741

105. Qin L, Selani D, Zegers F, et al. POS1091 Development and Validation of 'Rheumatic ' - A Digital Symptom Checker for Confirmation and Prediction of Rheumatic Diseases: A Large-Scale Prospective Study. *Annals of the Rheumatic Diseases* 2025;84(Supplement 1):1181EP-82. doi: https://dx.doi.org/10.1016/j.ard.2025.06.444

106. Qin L, Selani D, Zegers F, et al. Development and Validation of 'Rheumatic ' - A Digital Symptom Checker for Confirmation and Prediction of Rheumatic Diseases: A Large-Scale Prospective Study. *Annals of the Rheumatic Diseases* 2025;84(Supplement 1):1181EP-82. doi: https://dx.doi.org/10.1016/j.ard.2025.06.444

107. Ratzlaff C, Koehoorn M, Cibere J, et al. Clinical validation of an Internet-based questionnaire for ascertaining hip and knee osteoarthritis. *Osteoarthritis and Cartilage* 2012;20(12):1568-73. doi: 10.1016/j.joca.2012.08.017

108. Raza H, Rathee D, Amorim R, et al. Optimizing Patient Care Pathways: Impact Analysis of an AI-Assisted Smart Referral System for Musculoskeletal Services. *2024 IEEE International Conference on Digital Health (ICDH)* 2024:68-72. doi: 10.1109/ICDH62654.2024.00021

109. Redeker I, Tsiami S, Eicker J, et al. IDENTIFICATION OF A DIAGNOSTIC MODEL FOR AXIAL SPONDYLOARTHRITIS IN DAILY CLINICAL PRACTICE USING A RANDOM FOREST MACHINE LEARNING APPROACH. 2023;82(Supplement 1) doi: https://dx.doi.org/10.1136/annrheumdis-2023-eular.3030

110. Redeker I, Tsiami S, Eicker J, et al. Identification of a machine learning-based diagnostic model for axial spondyloarthritis in rheumatological routine care using a random forest approach. *RMD open* 2024;10(4) doi: https://dx.doi.org/10.1136/rmdopen-2024-004702

111. Roch FE, Hahn FM, Jackle K, et al. Diagnosis, treatment, and prevention of ankle sprains: Comparing free chatbot recommendations with clinical guidelines. *Foot and ankle surgery : official journal of the European Society of Foot and Ankle Surgeons* 2025;31(4):329-51. doi: https://dx.doi.org/10.1016/j.fas.2024.12.003

112. Roivainen P, Hoikka MJ, Raatiniemi L, et al. Telephone triage performed by nurses reduces non-urgent ambulance missions: A prospective observational pilot study in Finland. 2020;64(4) doi: https://dx.doi.org/10.1111/aas.13542

113. Ronicke S, Hirsch MC, Turk E, et al. Can a decision support system accelerate rare disease diagnosis? Evaluating the potential impact of Ada DX in a retrospective study. 2019;14(1) doi: https://dx.doi.org/10.1186/s13023-019-1040-6

114. Rostom K, Smith CD, Liddy C, et al. Improving Access to Rheumatologists: Use and Benefits of an Electronic Consultation Service. 2018;45(1) doi: https://dx.doi.org/10.3899/jrheum.161529

115. Ryan K, Grinbergs P. Analgesia use in people with low back pain self-referring into MSK care via Digital Triage in England. An observational Study. 2024;123(Supplement 1) doi: https://dx.doi.org/10.1016/j.physio.2024.04.262

116. Saif A, Wall A, Kang Y, et al. IMPROVING THE BIOPSYCHOSOCIAL ASSESSMENT AND MANAGEMENT OF PATIENTS WITH FIBROMYALGIA: DEVELOPING A DIGITAL PRE-CLINIC ASSESSMENT TOOL. 2023;62(Supplement 2) doi: https://dx.doi.org/10.1093/rheumatology/kead104.023

117. Salisbury C, Foster NE, Hopper C, et al. A pragmatic randomised controlled trial of the effectiveness and cost-effectiveness of 'PhysioDirect' telephone assessment and advice services for physiotherapy. 2013;17(2) doi: https://dx.doi.org/10.3310/hta17020

118. Salmeron JL, Rahimi SA, Navali AM, et al. Medical diagnosis of Rheumatoid Arthritis using data driven PSO-FCM with scarce datasets. 2017;232((Salmeron) Universidad Pablo de Olavide, Km. 1 Utrera road, Seville 41013, Spain(Salmeron) Universidad Autonoma de Chile, 5 Poniente 1670, Talca, Chile(Salmeron) University of Hradec Kralove, Rokitanskeho 62, 50003 Hradec Kralove, Czechia(Rahimi) Universi) doi: https://dx.doi.org/10.1016/j.neucom.2016.09.113

119. Santosa A, Tan T, Roslan N, et al. Chatbots in SINGAPORE-SCREENING and triaging: Use of a chatbot for screening of rheumatic conditions in a multi-ethnic Asian population. 2023;26(Supplement 1) doi: https://dx.doi.org/10.1111/1756-185X.14502

120. Scheibe MM, Imboden JB, Schmajuk G, et al. Efficiency gains for rheumatology consultation from an electronic referral system in a safety net health system. 2013;65(SUPPL. 10) doi: https://dx.doi.org/10.1002/art.38216

121. Selani D, Qin L, Zegers F, et al. PREDICTING FIBROMYALGIA OUTCOME FROM A RHEUMATIC-SPECIFIC SURVEY. 2024;83(Supplement 1) doi: https://dx.doi.org/10.1136/annrheumdis-2024-eular.2837

122. Sexton V, Atherton H, Dale J, et al. Clinician-led secondary triage in England's urgent care delivery: a cross-sectional study. 2023;73(731) doi: https://dx.doi.org/10.3399/BJGP.2022.0374

123. Shahid U, Meyer AN, Giardina T, et al. What misdiagnosed patients can tell us about types and origins of diagnostic errors. 2018;5(4) doi: https://dx.doi.org/10.1515/dx-2018-0095

124. Shapiro J, Getz B, Cohen SB, et al. Evaluation of a machine learning tool for the early identification of patients with undiagnosed psoriatic arthritis - A retrospective population-based study. 2023;7((Shapiro, Shovman) Maccabi Healthcare Services, Israel(Getz, Jenudi, Underberger, Dreyfuss, Ber, Steinberg-Koch) Predicta Med Analytics Ltd, Israel(Cohen) Metroplex Clinical Research Center, Dallas, TX, United States(Ben-Tov, Shovman) Maccabi Institute fo) doi: https://dx.doi.org/10.1016/j.jtauto.2023.100207

125. Shayo MJ, Shayo P, Haukila KF, et al. Expanding access to rehabilitation using mobile health to address musculoskeletal pain and disability. 2023;3 doi: 10.3389/fresc.2022.982175

126. Siebelt M, Das D, Van Den Moosdijk A, et al. Machine learning algorithms trained with pre-hospital acquired history-taking data can accurately differentiate diagnoses in patients with hip complaints. 2021;92(3) doi: https://dx.doi.org/10.1080/17453674.2021.1884408

127. Silva LB, Pereira DN, Chagas VS, et al. Orthopedic Asynchronous Teleconsultation for Primary Care Patients by a Large-Scale Telemedicine Service in Minas Gerais, Brazil. 2022;28(8) doi: 10.1089/tmj.2021.0293

128. Silverman SL, Calderon AD. The utility and limitations of FRAX: A us perspective. 2010;8(4) doi: https://dx.doi.org/10.1007/s11914-010-0032-1

129. Simmich J, Ross MH, Andrews NE, et al. Content and Quality of Mobile Apps for the Monitoring of Musculoskeletal or Neuropathic Pain in Australia: Systematic Evaluation. 2023;11 doi: 10.2196/46881

130. Singh P. Knowledge-base medical decision support system for knee pain management. 2013;4(4) doi: https://dx.doi.org/10.5958/j.0976-5506.4.4.169

131. Slattery B, Ackerman L, Jagadamma KC. Service evaluation of telehealth in a physiotherapy musculoskeletal setting: Patient outcomes and results from risk stratification. 2022;20(4) doi: https://dx.doi.org/10.1002/msc.1623

132. Spasic I, Button K. Patient Triage by Topic Modeling of Referral Letters: Feasibility Study. 2020;8(11) doi: https://dx.doi.org/10.2196/21252

133. Stanley AL, Edwards TC, Jaere MD, et al. An automated, web-based triage tool may optimise referral pathways in elective orthopaedic surgery: A proof-of-concept study. *DIGITAL HEALTH* 2023;9:20552076231152177. doi: 10.1177/20552076231152177

134. Torad AA, Ahmed MM, Elabd OM, et al. Identifying Predictors of Neck Disability in Patients with Cervical Pain Using Machine Learning Algorithms: A Cross-Sectional Correlational Study. 2024;13(7) doi: https://dx.doi.org/10.3390/jcm13071967

135. Turner J, Cathain A, Knowles E, et al. Impact of the urgent care telephone service NHS 111 pilot sites: a controlled before and after study. *BMJ Open* 2013;3(11):e003451. doi: 10.1136/bmjopen-2013-003451

136. Ullah KA, Rehman F, Anwar M, et al. Machine learning-based prediction of osteoporosis in postmenopausal women with clinical examined features: A quantitative clinical study. 2023;6(10) doi: https://dx.doi.org/10.1002/hsr2.1656

137. Umapathy H, Bennell K, Dickson C, et al. My joint pain: Web-based osteoarthritis management resource improves quality of care. 2015;45(Supplement 2) doi: https://dx.doi.org/10.1111/imj.12752

138. Umapathy H, Bennell K, Dickson C, et al. The Web-Based Osteoarthritis Management Resource My Joint Pain Improves Quality of Care: A Quasi-Experimental Study. *J Med Internet Res* 2015;17(7):e167. doi: 10.2196/jmir.4376

139. Valsecchi D, Moscatelli Spinelli F, Quattrina E, et al. RWD127 Preliminary Results of a Machine Learning Model for the Detection of Undiagnosed Cases of Ankylosing Spondylitis in Italy. *Value in Health* 2024;27(12 Supplement):S598. doi: https://dx.doi.org/10.1016/j.jval.2024.10.3685

140. Van Hooff M, Van Loon J, Van Limbeek J, et al. Development of a clinical decision tool to support spine surgeons in the triage of chronic lowback pain patients. 2013;22(5 SUPPL. 1) doi: https://dx.doi.org/10.1007/s00586-013-2947-6

141. van Hooff ML, van Loon J, van Limbeek J, et al. The Nijmegen decision tool for chronic low back pain. Development of a clinical decision tool for secondary or tertiary spine care specialists. 2014;9(8) doi: https://dx.doi.org/10.1371/journal.pone.0104226

142. Vardell E, Moore M. Isabel, a Clinical Decision Support System. *Medical Reference Services Quarterly* 2011;30(2):158-66. doi: 10.1080/02763869.2011.562800

143. Venerito V, Iannone F. Large language model-driven sentiment analysis for facilitating fibromyalgia diagnosis. 2024;10(2) doi: https://dx.doi.org/10.1136/rmdopen-2024-004367

144. Verzantvoort NCM, Teunis T, Verheij TJM, et al. Self-triage for acute primary care via a smartphone application: Practical, safe and efficient? *PLOS ONE* 2018;13(6):e0199284. doi: 10.1371/journal.pone.0199284

145. Vomer RP, 2nd, Lewno AJ, Udoh I, et al. Evaluation of the Hand and Wrist via Telephone and Video Visit. 2023;15(2) doi: https://dx.doi.org/10.7759/cureus.35322

146. Wallnofer A, Burgstaller JM, Weiss K, et al. Developing and testing a framework for coding general practitioners' free-text diagnoses in electronic medical records - a reliability study for generating training data in natural language processing. 2024;25(1) doi: https://dx.doi.org/10.1186/s12875-024-02514-1

147. Wathall S, Foster N, Hill J, et al. Health informatics (HI) innovations in randomised trials and clinical cohorts-identification, screening, stratified care and data collection during primary care consultations. 2019;20 doi: https://doi.org/10.1186/s13063-019-3688-6

148. Weisberg EM, Chu LC, Fishman EK. The first use of artificial intelligence (AI) in the ER: triage not diagnosis. 2020;27(4) doi: https://dx.doi.org/10.1007/s10140-020-01773-6

149. Westley S, Mistry R, Dheansa B. Accuracy of virtual assessment in hand trauma. 2022;31((Westley, Mistry, Dheansa) The Department of Plastic Surgery, The Queen Victoria Hospital, Holtye Road, East Grinstead RH19 3DZ, United Kingdom) doi: https://dx.doi.org/10.1016/j.jpra.2021.10.008

150. Willmore E, Donachie P, Van der Windt D, et al. Musculoskeletal triage: Do advanced practitioners agree on referral allocation?...Physiotherapy UK Virtual Conference, November 5-6, 2021.114 doi: 10.1016/j.physio.2021.12.112

151. Wu K-T, Chen P-C, Chou W-Y, et al. Diagnostic Accuracy and Interobserver Reliability of Rotator Cuff Tear Detection With Ultrasonography Are Improved With Attentional Deep Learning. *Arthroscopy : the journal of arthroscopic & related surgery : official publication of the Arthroscopy Association of North America and the International Arthroscopy Association* 2025;41(8):2708-16. doi: https://dx.doi.org/10.1016/j.arthro.2024.12.024

152. Yildiz NT, Kocaman H, Yildirim H, et al. An investigation of machine learning algorithms for prediction of temporomandibular disorders by using clinical parameters. *Medicine* 2024;103(41):e39912. doi: https://dx.doi.org/10.1097/MD.0000000000039912

153. Yu D, Peat G, Bedson J, et al. Weighted cumulative exposure models helped identify an association between early knee-pain consultations and future knee OA diagnosis. 2016;76(jce, 8801383) doi: https://dx.doi.org/10.1016/j.jclinepi.2016.02.025

154. Zatt FP, Cordeiro JVC, Bohner L, et al. Using machine learning to classify temporomandibular disorders: a proof of concept. *Journal of applied oral science : revista FOB* 2024;32(101189774):e20240282. doi: https://dx.doi.org/10.1590/1678-7757-2024-0282

155. Zegers F, Qin L, Selani D, et al. POS1131 Prediction Models for Rheumatic Diseases: From Clinical Simplicity to Data-Driven Complexity with Patient-reported Symptoms for an Online Symptom Checker. *Annals of the Rheumatic Diseases* 2025;84(Supplement 1):1211EP-12. doi: https://dx.doi.org/10.1016/j.ard.2025.06.481

156. Zhou Y, Moon C, Szatkowski J, et al. Evaluating ChatGPT responses in the context of a 53-year-old male with a femoral neck fracture: a qualitative analysis. 2024;34(2) doi: https://dx.doi.org/10.1007/s00590-023-03742-4

157. Ziabari MK, Amyot D, Michalowski W, et al. Creating Mobile Self-Triage Applications: Requirements and Usability Perspectives. *2021 IEEE 29th International Requirements Engineering Conference Workshops (REW)* 2021:268-77. doi: 10.1109/REW53955.2021.00048
