# Supplementary material for: Evidence for Digital Health Tools Designed to Support the Triage of Musculoskeletal Conditions in Primary, Urgent, and Emergency Care Settings: Scoping Review
Source: J Med Internet Res. 2026 Jan 14;28:e81578. doi: 10.2196/81578 (PMC12803503; doi:10.2196/81578)
Supplement: Multimedia Appendix 4 [file jmir-v28-e81578-s004.docx]

| Appendix 4. Characteristics of included studies, summarizing design, demographics, and digital tool features. | | | | | | | |  |
| --- | --- | --- | --- | --- | --- | --- | --- | --- |
| Author  Year, Country | Purpose | Study design and Study Time Frame | Study Population,  Sample size  (Disease Studied)^1^ | Age (yrs) (mean, range), Sex (% female)^2^ | Digital health tool studied^3^ | Care setting  of study^4^ | Intended user for tool |  |
| Badahman et al 2024,[28]  Saudi Arabia | Assess the diagnostic precision of AI as a CDSS in comparison to MRI for diagnosing patients with lumbar disc herniation. | multi-methods  (peer reviewed)  10/2023-01/2024 | MSK  n=100  (100% low back pain) | 48.25(18-84)  F:NR, M:NR | Therapha | ED | Clinician |  |
| Badsha et al 2024,[29]  United Arab Emirates | Evaluate consecutive new referrals to a rheumatology clinic both by rheumatologists and by Proprietary Rule Engine and AI GPT4. | cross-sectional  (abstract)  10/2023-01/2024 | MSK  n=100  (100% rheumatoid arthritis) | NR  F:NR, M:NR | Chat GPT/Rule Engine | Primary care (physician-led offices) | Patient |  |
| Bisson et al 2014,[30]  United States | Design and evaluate an internet-based program that generates a differential diagnosis based on a history of knee pain entered by the patient. | non-randomized intervention  (peer-reviewed)  12/2012-03/2013 | MSK  n=527  (100% knee injuries) | 47 (18-84)  F=255, M=272 (48%) | Virtual  Knee Doc | Primary care (physician-led offices) | Patient |  |
| Bisson et al 2016,[31]  United States | Determine patients’ ability to select their diagnosis from a list of possible diagnoses supplied by an accurate symptom checker. | cross-sectional  (peer-reviewed)  06/2014-01/2015 | MSK  n=328  (100% knee injuries) | 48 (18-76)  F=165, M=163 (50%) | Virtual  Knee Doc | Primary care (physician-led offices) | Patient |  |
| Bond et al 2024,[32]  United Kingdom | Introduce the details within the TT (digital triage) and ST (self-management rehab application) and explore a patient journey, outcome and user feedback. | case report  (grey literature)  2022 | MSK  n=1  (100% MSK) | 46  F=1, M=0 (100%) | Phio | Self-access | Patient |  |
| Carmona et al 2022,[33]  United States | Address methodological limitations of prior information-seeking research and examine who seeks information from an intelligent web-based symptom checker and for what purpose, how users experience the tool, what they intend to do with the information, and predictors of intentions to follow the tool recommendations. | cross-sectional  (peer-reviewed)  01/2019-02/2019 | Generic conditions with MSK  n=2437  (35% MSK) | 39.35 (18-87)  F=2069, M=368 (85%) | Buoy  Health | Self-access | Patient |  |
| Daher et al 2023,[34]  United States | Investigate the capabilities and implications of ChatGPT in diagnosing and managing shoulder and elbow complaints in a private clinic setting. | cross-sectional  (peer-reviewed)  NR | MSK  n=29  (100% shoulder and elbow injuries) | NR  F=14, M=15 (48%) | Chat GPT | Primary care (physician-led offices) | Patient |  |
| Demmelmaier et al 2010,[35]  Sweden | Evaluate the interrater reliability of a research protocol designed to analyze screening of physical and psychosocial risk factors for prolonged disability in back pain during telephone consultations | cross-sectional  (peer-reviewed)  2007-2008 | MSK  n=100  (100% low back pain) | NR  F:NR, M:NR | NR | Primary Care  (physiotherapist-led offices) | Clinician |  |
| Dias et al 2012,[36]  NR | Assess whether emergency clinic appointments could be avoided by implementing a telephone-based pathway using patient self-assessment of nasal injuries. | quasi-experimental  (abstract)  NR | MSK  n=67  (100% nasal injuries) | NR  F:NR, M:NR | NR | ED | Clinician |  |
| Graf et al 2022,[37]  Germany | Compare the diagnostic accuracy of an AI-based symptom checker app (Ada) and physicians regarding the presence/absence of an IRD. | cross-sectional  (peer-reviewed)  NR | MSK  n=20  (100% rheumatic and MSK conditions) | 53.50 (27-76)  F=16, M=4 (80%) | ADA | Self-access | Patient |  |
| Gomez-Centeno et al 2025,[38]  Spain | Evaluate the effectiveness of ReumAI, an AI-based triage tool, in streamlining rheumatology consultations within a private hospital setting. | quasi-experimental  (abstract)  NR | MSK  n=300  (100% rheumatoid arthritis) | 52  F=210, M=90 (70%) | ReumAI | Self-access | Patient |  |
| Gymer et al 2023,[39]  United Kingdom | Introduce the details within the TT (digital triage) and ST (self-management rehab application) and explore a patient journey, outcome and user feedback. | case report  (grey literature)  01/2022 | MSK  N=1  (100% MSK | 60  F=0, M=1 (0%) | Phio | Self-access | Patient |  |
| Hageman et al 2015,[40]  United States | Test the null hypothesis that there were no factors associated with correspondence between online diagnosis and the hand surgeon’s diagnosis in an outpatient hand and upper extremity surgeon’s office | cross-sectional  (peer-reviewed)  NR | MSK  n=86  (100% hand injuries) | 46  F:NR, M:NR | WebMD Symptom Checker | Self-access | Both |  |
| Hannah et al 2024,[41]  Germany | Investigate an asynchronous telediagnostic approach in patients with suspected axSpA. | quasi-experimental  (peer-reviewed)  NR | MSK  n=36  (100% Axial spondylo-arthropathy) | 37.19^†^  F=21, M=15 (58%) | Bechterew-check  ADA | Self-access | Urban |  |
| Hara et al2015,[[42]  Japan | Investigate triage of transportation of patients with severe finger injuries in the city of Nagoya from 2010 to 2013 using an Interactive Teletriage. | quasi-experimental  (peer-reviewed)  2010-2013 | Generic conditions with MSK n=45  (100% finger injuries) | NR | Phone Camera | ED | Clinician |  |
| Jakobi et al 2025,[43]  Germany | Investigate usability and user acceptance of Rheumatic? with both quantitative and qualitative means in a real-world environment of an outpatient rheumatology clinic | Retrospective  (peer-reviewed)  06/2023-04/2024 | MSK  n=105  (100% rheumatoid arthritis) | 50 (34-76)  F=68, M=37 (65%) | Rheumatic? | Self-access | Patient |  |
| Kelly et al 2021,[44]  Ireland | Evaluate whether a telephone assessment and advice service can reduce the wait time and non-attendance rate for physiotherapy compared to the usual care pathway. | non-randomized intervention  (peer-reviewed)  05-08/2018, 05/2019 | MSK  n=116  (100% MSK) | 42^†^ (31-66)  F=43, M=60 (37%) | PhysioDirect | ED | Clinician |  |
| Knevel et al 2022,[45]  Sweden, Germany, Netherlands | Test a multilingual, comprehensive DDSS in individuals whom rheumatic disease is suspected, to analyze the discriminatory ability between patients with and without immune-mediated rheumatic diseases. | retrospective cohort  (peer-reviewed)  NR | MSK  n=174  (100% inflammatory arthritis) | 54**^‡^** (19-84)  F=117, M=57 (67%) | Rheumatic? | Self-access | Patient |  |
| Knitza et al 2021,[46]  Germany | Create real world-based evidence by evaluating the diagnostic accuracy, usability, acceptability, and completion time of two free, publicly available symptom checkers, Ada and Rheport. | randomized controlled trial (peer-reviewed)  09/2019-02/2020 | MSK  n=164  (100% inflammatory arthritis) | 51^†^ (38.8-61)^§^  F=113, M=51 (69%) | Rheport, ADA | Self-access | Patient |  |
| Knitza et al 2022,[47]  Germany | Evaluate patient’s previous online assessment experiences, to compare the acceptability, usability, usefulness and potential impact of AI-based symptom checker (Ada) and an online questionnaire based self-referral tool (Rheport) using the final dataset from all three rheumatology centers. | randomized controlled trial (peer-reviewed)  09/2019-04/2021 | MSK  n=600  (100% inflammatory arthritis) | 52^†^ (37-61)^§^  F=418, M=182 (70%) | Rheport, ADA | Self-access | Patient |  |
| Knitza et al 2024,[48]  Germany | Evaluate the diagnostic capability of Ada and Rheport in identifying IRDs. | randomized controlled trial (peer-reviewed)  09/2019-04/2021 | MSK  n=600  (100% inflammatory arthritis) | 52^†^ (37-61)^§^  F=418, M=182 (70%) | Rheport, ADA | Self-access | Patient |  |
| Krusche et al 2023, [49]  Germany | Assess the diagnostic accuracy of  ChatGPT-4 in comparison to a previous analysis including physicians and symptom checker (ADA) regarding rheumatic and  musculoskeletal diseases. | cross-sectional  (peer-reviewed)  NR | MSK  n=20  (100% rheumatic and MSK conditions) | 53.50 (27-76)  F=16, M=4 (80%) | ChatGPT  ADA | Self-access | Patient |  |
| Li et al 2023,[50]  United States | Evaluate a Veterans Affairs (VA) tele–emergency care (tele- EC) pilot aimed at reducing reliance on out-of- network ED care, a growing portion of VA spending. With this service, an emergency physician virtually evaluated selected veterans calling a nurse triage line. | non-randomized intervention  (peer-reviewed)  01/2021-12/2021 | Generic conditions with MSK  n=2132  (27% MSK) | NR | TriageXpert Dual Purpose | Urgent Care | Both |  |
| Lowe et al 2022,[51]  United Kingdom | Maximize usability before evaluating the safety and effectiveness of DART through a randomized controlled trial, the pilot protocol for which has been published | mixed methods  (peer-reviewed)  NR | MSK  n=22  (100% MSK) | 48.6 (20-77)  F:NR, M:NR | Digital Assessment Routing tool (DART) | Self-access | Patient |  |
| Lowe et al 2024,[52]  United Kingdom | Evaluate trial design, assess procedures, and collect exploratory data to assess the feasibility of delivering an adequately powered, definitive crossover noninferiority randomized trial, assessing DART safety and efficacy in an NHS primary care setting. | randomized controlled trial (peer-reviewed)  NR | MSK  n=78  (100% MSK) | 52.9 (18-78)  F:NR, M:NR | Digital Assessment Routing tool (DART) | Self-access | Patient |  |
| Lundberg et al 2023,[53]  Netherlands,  United Kingdom | Differentiate patients with and without autoimmune inflammatory rheumatological musculoskeletal diseases. | prospective cohort  (peer-reviewed)  07/2021-11/2022 | MSK  n=12,712  (100% inflammatory arthritis) | NR | Rheumatic? | Self-access | Both |  |
| Martin and Payne 2020,[54]  UK | Explore if a digital physiotherapy self-referral service is safe and acceptable to patients with back pain. | mixed methods (abstract) | MSK  n=22  (100% low back pain) | NR | NR | Self-access | Patient |  |
| Phillips et al 2012,[55]  United Kingdom | Report on the evaluation of the programme pilot, (physiotherapy service provided by three NHS Trusts in Wales–Hywel Dda, Gwent and Northwest Wales) focussing on feasibility and cost-effectiveness. | non-randomized intervention  (peer-reviewed)  09/2008-02/2009 | MSK  n=514  (100% MSK) | NR | Bespoke tool | Self-access,  Primary Care (physiotherapist-led offices) | Both |  |
| Qin et al 2024,[56]  Netherlands | Assess the differences in Rheumatic? total scores between individuals diagnosed with IRD and non-IRD. | prospective cohort  (abstract)  07/2021-07/2022 | MSK  n=8,727  (100% inflammatory arthritis) | NR | Rheumatic? | Self-access | Patient |  |
| Ryan and Grinbergs 2024,[57]  United Kingdom | Report on an innovative, patient-facing digital triage (DT), to facilitate immediate self-referral for those wanting to access MSK physiotherapy services with rapid onward referral or signposting to the appropriate care pathway | retrospective cohort  (abstract)  02/2022-08/2022 | MSK  n=4,627  (100% MSK) | NR | NR | Self-access | Patient |  |
| Salisbury et al 2013,[58]  United Kingdom | Assess the clinical effectiveness and cost effectiveness of PhysioDirect compared with usual models of care based on patients joining a waiting list for physiotherapy and eventually receiving face-to-face care. | randomized controlled trial (peer-reviewed)  07/2009-12/2009 | MSK  n=2,249  (100% MSK) | 48 (36-62)  F:NR, M:NR | PhysioDirect | Self-access | Clinician |  |
| Soin et al 2022,[59]  United States | Evaluate whether it is possible to use AI/ML to analyze specific data points and to predict the most likely diagnosis related to spinal pain. | cross-sectional  (peer-reviewed)  NR | MSK  n=246  (100% low back pain) | 57.4 (18-91)  F:NR, M:NR | Bespoke  tool | Primary Care (physician-led offices) | Clinician |  |
| Tan et al 2023,[60]  Singapore | Test the user acceptance of a comprehensive digital diagnostic decision support systems tailored for a multi-ethnic Asian population. | cross-sectional  (abstract)  NR | MSK  n=NR  (100% rheumatoid arthritis) | NR | Rheum  Connect | Self-access | Patient |  |
| Trivedi et al 2024,[61]  Canada | Assess the level of concordance between sets of self-triage scores and the reference standard of the triage score assigned by the usual process of nurse driven triage. | retrospective cohort  (peer-reviewed)  11/2019-03/2020 | Generic conditions with MSK  n=223  (41% MSK) | NR  F:NR, M:NR | Bespoke  tool | ED | Patient |  |
| ED=emergency department; NA=not applicable; NR=not reported; MSK=musculoskeletal  ^1^Study population was categorized into 2 groups: MSK= study focused on MSK conditions; Generic conditions with MSK=study focused on general health conditions with a subgroup consisting of MSK conditions  If study focused on a specific condition/disease, the percentage of this was also reported alongside the sample size  ^2^Age reported as mean unless reported: ^†^median reported; ^§^range reported as IQR; ^‡^median of means  ^3^If tool name was not reported, we used the label ‘bespoke tool’  ^4^Care setting represents the environment in which the tool was tested or implemented:  ED=hospital setting that provides life- and limb-saving care  Primary care=first point-of-contact care (outside the ED) and includes physician-led (general practitioner or specialist clinics) and physiotherapist-led clinics (seen by a physiotherapist first)  Self-access=patient can access tool independent of seeing a health professional | | | | | | | | |

**REFERENCES**

28. Badahman F, Alsobhi M, Alzahrani A, Chevidikunnan MF, Neamatallah Z, Alqarni A, et al. Validating the Accuracy of a Patient-Facing Clinical Decision Support System in Predicting Lumbar Disc Herniation: Diagnostic Accuracy Study. Diagnostics. 2024;14(17):1870. PMID: 2031340140. doi: <https://dx.doi.org/10.3390/diagnostics14171870>.

29. Badsha HM, Khan B, Harifi G, Ja A, Raman S. IS THE FUTURE OF RHEUMATOLOGY HERE A STUDY OF A PROPRIETARY RULE ENGINE AND ARTIFICIAL INTELLIGENCE GPT4 (AI GPT4) FOR INITIAL EVALUATION OF RHEUMATOLOGY CASES. 2024;83(Supplement 1). PMID: 644868572. doi: <https://dx.doi.org/10.1136/annrheumdis-2024-eular.1942>.

30. Bisson LJ, Komm JT, Bernas GA, Fineberg MS, Marzo JM, Rauh MA, et al. Accuracy of a computer-based diagnostic program for ambulatory patients with knee pain. 2014;42(10). doi: <https://dx.doi.org/10.1177/0363546514541654>.

31. Bisson LJ, Komm JT, Bernas GA, Fineberg MS, Marzo JM, Rauh MA, et al. How Accurate Are Patients at Diagnosing the Cause of Their Knee Pain With the Help of a Web-based Symptom Checker? Orthopaedic Journal of Sports Medicine. 2016;4(2):2325967116630286. doi: 10.1177/2325967116630286.

32. Bond C, Guard M, Grinbergs P. Case report: Digital Musculoskeletal triage and rehabilitation tools enhance accessibility, user experience and outcomes in mechanical knee pain. 2024;123(Supplement 1). PMID: 2032869206. doi: <https://dx.doi.org/10.1016/j.physio.2024.04.143>.

33. Carmona KA, Chittamuru D, Kravitz RL, Ramondt S, Ramirez AS. Health Information Seeking From an Intelligent Web-Based Symptom Checker: Cross-sectional Questionnaire Study. 2022;24(8). PMID: 2019967973. doi: <https://dx.doi.org/10.2196/36322>.

34. Daher M, Koa J, Boufadel P, Singh J, Fares MY, Abboud JA. Breaking barriers: can ChatGPT compete with a shoulder and elbow specialist in diagnosis and management? 2023;7(6). PMID: 2027701830. doi: <https://dx.doi.org/10.1016/j.jseint.2023.07.018>.

35. Demmelmaier I, Denison E, Lindberg P, Asenlof P. Physiotherapists' telephone consultations regarding back pain: a method to analyze screening of risk factors. 2010;26(7). doi: <https://dx.doi.org/10.3109/09593980903433938>.

36. Dias L, Maughan E, Kisha A, Moorthy R. Telephone triage in the management of patients with nasal injuries. 2012;37(SUPPL. 1). PMID: 71023181. doi: <https://dx.doi.org/10.1111/j.1749-4486.2012.02517.x>.

37. Graf M, Knitza J, Leipe J, Krusche M, Welcker M, Kuhn S, et al. Comparison of physician and artificial intelligence-based symptom checker diagnostic accuracy. 2022;42(12). PMID: 2019061823. doi: <https://dx.doi.org/10.1007/s00296-022-05202-4>.

38. Gomez-Centeno A, Sabaris-Vilas M, Garcia-Sancho F, Segura-Sanchez J. POS0883 OPTIMIZING RHEUMATOLOGY CONSULTATIONS WITH ARTIFICIAL INTELLIGENCE: INSIGHTS FROM THE ReumAI PILOT STUDY. Annals of the Rheumatic Diseases. 2025;84(Supplement 1):1018. PMID: 2039187479. doi: <https://dx.doi.org/10.1016/j.ard.2025.06.238>.

39. Gymer M GM, Bringbergs P. Case Report: Digital Musculoskeletal Triage and Rehabilitation

Tools Improve Outcomes and Offer a Positive Experience for Lower Back Pain. Phio; 2023.

40. Hageman MGJS, Anderson J, Blok R, Bossen JKJ, Ring D. Internet Self-Diagnosis in Hand Surgery. HAND. 2014;10(3):565-9. doi: 10.1007/s11552-014-9707-x.

41. Hannah L, von Sophie R, Gabriella RM, Daniela B, Harriet M, Britta H, et al. Stepwise asynchronous telehealth assessment of patients with suspected axial spondyloarthritis: results from a pilot study. Rheumatology International. 2024;44(1):173-80. doi: 10.1007/s00296-023-05360-z.

42. Hara T, Nishizuka T, Yamamoto M, Iwatsuki K, Natsume T, Hirata H. Teletriage for patients with traumatic finger injury directing emergency medical transportation services to appropriate hospitals: A pilot project in Nagoya City, Japan. 2015;46(7). PMID: 603205702. doi: <https://dx.doi.org/10.1016/j.injury.2015.02.022>.

43. Jakobi S, Boy K, Wagner M, May S, Temiz A, Liphardt AM, et al. Rheumatic? A diagnostic decision support tool for individuals suspecting rheumatic diseases: Mixed-methods usability and acceptability study. BMC Rheumatology. 2025;9(1):59. PMID: 2034665973. doi: <https://dx.doi.org/10.1186/s41927-025-00507-w>.

44. Kelly M, Higgins A, Murphy A, McCreesh K. A telephone assessment and advice service within an ED physiotherapy clinic: a single-site quality improvement cohort study. Archives of Physiotherapy. 2021;11(1). doi: 10.1186/s40945-020-00098-4.

45. Knevel R, Knitza J, Hensvold A, Circiumaru A, Bruce T, Evans S, et al. Rheumatic?-A Digital Diagnostic Decision Support Tool for Individuals Suspecting Rheumatic Diseases: A Multicenter Pilot Validation Study. 2022;9((Knevel, Maarseveen, Maurits, Beaart-van de Voorde, Huizinga) Leiden University Medical Center, Leiden, Netherlands(Knevel) Translational and Clinical Research Institute, Newcastle University, Newcastle upon Tyne, United Kingdom(Knitza, Simon, Kleyer, Sch). PMID: 2016520483. doi: <https://dx.doi.org/10.3389/fmed.2022.774945>.

46. Knitza J, Mohn J, Bergmann C, Kampylafka E, Hagen M, Bohr D, et al. Accuracy, patient-perceived usability, and acceptance of two symptom checkers (Ada and Rheport) in rheumatology: interim results from a randomized controlled crossover trial. Arthritis Research & Therapy. 2021;23(1):112. doi: 10.1186/s13075-021-02498-8.

47. Knitza J, Muehlensiepen F, Ignatyev Y, Fuchs F, Mohn J, Simon D, et al. Patient's Perception of Digital Symptom Assessment Technologies in Rheumatology: Results From a Multicentre Study. 2022;10((Knitza, Fuchs, Mohn, Simon, Kleyer, Fagni, Boeltz, Morf, Bergmann, Labinsky, Ramming, Distler, Schett) Department of Internal Medicine 3, Erlangen, Germany(Knitza, Fuchs, Mohn, Simon, Kleyer, Fagni, Boeltz, Morf, Bergmann, Labinsky, Ramming, Distler, Sch). PMID: 637486321. doi: <https://dx.doi.org/10.3389/fpubh.2022.844669>.

48. Knitza J, Tascilar K, Fuchs F, Mohn J, Kuhn S, Bohr D, et al. Diagnostic Accuracy of a Mobile AI-Based Symptom Checker and a Web-Based Self-Referral Tool in Rheumatology: Multicenter Randomized Controlled Trial. 2024;26((Knitza, Kuhn) Institute for Digital Medicine, University Hospital Giessen-Marburg, Philipps University Marburg, Marburg, Germany(Knitza, Muehlensiepen, Vuillerme) AGEIS, Universite Grenoble Alpes, Grenoble, France(Knitza, Tascilar, Fuchs, Mohn, Bohr, Ber). PMID: 2033486993. doi: <https://dx.doi.org/10.2196/55542>.

49. Krusche M, Callhoff J, Knitza J, Ruffer N. Diagnostic accuracy of a large language model in rheumatology: comparison of physician and ChatGPT-4. Rheumatology International. 2023;44(2):303-6. doi: 10.1007/s00296-023-05464-6.

50. Li KY, Kim PS, Thariath J, Wong ES, Barkham J, Kocher KE. Standard nurse phone triage versus tele-emergency care pilot on Veteran use of in-person acute care: An instrumental variable analysis. 2023;30(4). PMID: 2021979976. doi: <https://dx.doi.org/10.1111/acem.14681>.

51. Lowe C, Browne M, Marsh W, Morrissey D. Usability Testing of a Digital Assessment Routing Tool for Musculoskeletal Disorders: Iterative, Convergent Mixed Methods Study. J Med Internet Res. 2022;24(8):e38352. doi: 10.2196/38352.

52. Lowe C, Sephton R, Marsh W, Morrissey D. Evaluation of a Musculoskeletal Digital Assessment Routing Tool (DART): Crossover Noninferiority Randomized Pilot Trial. JMIR Form Res. 2024;8:e56715. doi: 10.2196/56715.

53. Lundberg K, Qin L, Aulin C, van Spil WE, Maurits MP, Knevel R. Population-based user-perceived experience of &lt;em&gt;Rheumatic?&lt;/em&gt;: a novel digital symptom-checker in rheumatology. RMD Open. 2023;9(2):e002974. doi: 10.1136/rmdopen-2022-002974.

54. Martin MJ, Payne KM. Using digital technology and user-centred design to develop a physiotherapy self-referral service for back pain. 2020;107(Supplement 1). PMID: 2006783802. doi: <https://dx.doi.org/10.1016/j.physio.2020.03.203>.

55. Phillips CJ, Phillips R, Main CJ, Watson PJ, Davies S, Farr A, et al. The cost effectiveness of NHS physiotherapy support for occupational health (OH) services. 2012;13. PMID: WOS:000305613700001. doi: 10.1186/1471-2474-13-29.

56. Qin L, Zegers F, Selani D, Van Den Akker EB, Bos R, Le Cessie S, et al. DIFFERENTIATION OF IMMUNE MEDIATED VERSUS NON IMMUNE MEDIATED RHEUMATIC DISEASES BY ONLINE SYMPTOM CHECKER IN REALWORLD PATIENTS - MULTIPLE DIAGNOSES AND PARTICULARLY FIBROMYALGIA IS A STUMBLING BLOCK. 2024;83(Supplement 1). PMID: 644868620. doi: <https://dx.doi.org/10.1136/annrheumdis-2024-eular.5438>.

57. Ryan K, Grinbergs P. Demographic analysis of users of a musculoskeletal physiotherapy self-referral digital triage tool in Bromley. Physiotherapy. 2024;123:e210-e1. doi: 10.1016/j.physio.2024.04.263.

58. Salisbury C, Foster NE, Hopper C, Bishop A, Hollinghurst S, Coast J, et al. A pragmatic randomised controlled trial of the effectiveness and cost-effectiveness of 'PhysioDirect' telephone assessment and advice services for physiotherapy. 2013;17(2). doi: <https://dx.doi.org/10.3310/hta17020>.

59. Soin A, Hirschbeck M, Verdon M, Manchikanti L. A Pilot Study Implementing a Machine Learning Algorithm to Use Artificial Intelligence to Diagnose Spinal Conditions. 2022;25(2).

60. Tan T, Santosa A, Roslan N, Li J. The development of an AI-based conversational agent for screening of rheumatic diseases. 2023;26(Supplement 1). PMID: 640242766. doi: <https://dx.doi.org/10.1111/1756-185X.14505>.

61. Trivedi SV, Batta R, Henao–Romero N, Mondal P, Wilson T, Stempien J. A comparison of self-triage tools to nurse driven triage in the emergency department. PLOS ONE. 2024;19(8):e0297321. doi: 10.1371/journal.pone.0297321.
