# Supplementary material for: Evidence for Digital Health Tools Designed to Support the Triage of Musculoskeletal Conditions in Primary, Urgent, and Emergency Care Settings: Scoping Review
Source: J Med Internet Res. 2026 Jan 14;28:e81578. doi: 10.2196/81578 (PMC12803503; doi:10.2196/81578)
Supplement: Multimedia Appendix 5 [file jmir-v28-e81578-s005.docx]

| Tool name | Population Target | Interface | Reference |
| --- | --- | --- | --- |
| BONECheck | Frail, Aging | App | <https://www.medrxiv.org/content/10.1101/2023.05.10.23289825v1> |
| My Joint Pain | Osteoarthritis | Web-based | <https://www.myjointpain.org.au/> |
| Selfback | Back pain | App | <https://pubmed.ncbi.nlm.nih.gov/35130898/> |
| getUbetter | MSK | App | <https://www.getubetter.com/> |
| mySCS | Chronic pain | App | <https://www.pain.com/en/personal-support-resources/tools-you-can-use/pain-management-apps/myscs-app.html> |
| Smart triage | Paediatrics | App | <https://www.bcchr.ca/globalhealth/our-projects/project-highlights/smart-triage> |
| TriageGO | Generic Health + MSK | AI | <https://www.beckmancoulter.com/products/clinical-decision-support/triagego> |
| Salaso MSK digital triage | Generic MSK | AI | <https://salaso.com/msk-digital-triage/> |
| E-triage | Osteoporosis | Not reported | <https://doi.org/10.1007/s11657-020-0703-1> |
| Tele-triage/STarTBack | Back pain | Telephone | <https://onlinelibrary.wiley.com/doi/10.1002/msc.1623> |
